# TASC: a time-aware sequence clustering framework with uncertainty quantification for electronic health record trajectories

**DOI:** 10.1186/s13040-026-00569-7

**Published:** 2026-06-04

**Authors:** April Yujie Yan, Thomas K. M. Cudjoe, Casey Overby Taylor

**Affiliations:** 1 Department of Biomedical Engineering, Johns Hopkins School of Medicine, 214 Hackerman Hall, 3101 Wyman Park Dr, Baltimore, MD 21218 USA; 2 Institute for Computational Medicine, Johns Hopkins Whiting School of Engineering, 214 Hackerman Hall, 3101 Wyman Park Dr, Baltimore, MD 1218 USA; 3 Division of Geriatric Medicine and Gerontology, Johns Hopkins University School of Medicine, Baltimore, MD USA; 4 Division of General Internal Medicine, Department of Medicine, Johns Hopkins University School of Medicine, Baltimore, MD USA

**Keywords:** Longitudinal clustering, Electronic health records, Musculoskeletal disorders, Total knee replacement, Machine learning, Association analysis, Pattern mining

## Abstract

Longitudinal electronic health record (EHR) trajectories are highly heterogeneous, sparse, and irregular, making unsupervised temporal pattern discovery and uncertainty quantification challenging. We developed a Time-Aware Sequence Clustering (TASC) framework that integrates temporal spacing, clinical semantic similarity, and probabilistic subtype characterization to identify longitudinal trajectory patterns from EHR data. We demonstrate the framework using pre-surgical musculoskeletal care trajectories preceding total knee replacement (TKR). As a proof-of-concept application, we applied TASC to EHR and survey data from 2,052 patients undergoing primary TKR in the *All of Us* Research Program. Using electronic health records and survey data, we constructed their temporally ordered sequences of musculoskeletal-related diagnoses and comorbidities. We applied a weighted edit-distance algorithm that incorporates both clinical semantic similarity and inter-event time gaps, followed by K-Medoids clustering and subsampling-based stability analysis. Clusters were compared across sociodemographic variables, quality-of-life measures, prevalence of musculoskeletal-related diagnostic events, time to surgery, and pre- and post-surgical opioid use. We further quantified cluster membership uncertainty by computing probability-based subtype assignments from distances between partial or full trajectories and cluster medoids using a SoftMax transformation. TASC identified stable and interpretable trajectory structures from heterogeneous longitudinal EHR data in patients undergoing TKR. A five-cluster solution achieved the best silhouette score of 0.22 and demonstrated good stability under subsampling with mean Adjusted Rand Index of 0.71. The resulting trajectory clusters demonstrated distinct sociodemographic profiles, diagnostic/comorbidity patterns, and time-to-surgery distributions, supporting the framework’s ability to capture heterogeneous longitudinal care-utilization patterns. Patients with fast progression (median = 252 days) to surgery were younger (mean = 53.6 years, SD =16.5 years) and had minimal comorbidity, whereas joint pain–obesity (median = 766 days) and joint pain–fracture (median = 1154 days) subtypes showed intermediate progression timelines. The soft-tissue–dominant subtype was characterized by prolonged pre-surgical trajectories (median = 2900 days) and elevated chronic opioid use, while the complex, chronic subtype comprised older patients (mean = 62.7 years, SD = 10.0 years) with multimorbidity, long delays to surgery (median = 5457 days), and the lowest post-surgical quality-of-life scores. Opioid use declined after surgery across all clusters, with the largest reductions observed in clusters with less complexity and shorter pre-surgical trajectories (e.g., fast progression to surgery and joint pain-obesity subtypes, 38% and 31% reduction, respectively). Probability-based analysis showed that fast progression subtype was identifiable early and with high confidence, joint pain-obesity and joint pain-fracture subtypes reflected risk patterns already present in earlier musculoskeletal history, whereas soft-tissue–dominant and complex chronic subtypes required longer longitudinal care records to become distinguishable. TASC provides a flexible framework for uncertainty-aware clustering of longitudinal EHR trajectories and demonstrates how temporal and semantic sequence information can reveal heterogeneous care-utilization patterns in musculoskeletal disease. These findings support future applications of TASC to other longitudinal clinical domains and event representations.

**Supplementary Information:**

The online version contains supplementary material available at 10.1186/s13040-026-00569-7.

## Introduction

Longitudinal electronic health record (EHR) data contain complex temporal patterns that may reflect heterogeneous disease progression, healthcare utilization, and care-seeking behavior across patients [1–5]. However, Longitudinal EHR trajectories present major challenges for unsupervised temporal pattern discovery because clinical events are heterogeneous, sparse, irregularly timed, and often semantically overlapping [2, 3]. Musculoskeletal disorders (MSD), particularly pathways leading to total knee replacement (TKR), provide an important use case for studying such longitudinal care trajectories because patients often experience diverse diagnostic histories, comorbidities, and treatment patterns prior to surgery [6, 7].

Musculoskeletal disorders are among the leading causes of disability worldwide, and advanced disease often leads to joint replacement surgeries such as TKR [6, 7]. TKR, a form of arthroplasty in which damaged joint structures are replaced with a prosthesis, is widely performed to relieve pain and restore function in patients with end-stage knee osteoarthritis or related degenerative conditions [8]. The demand for TKR continues to increase, with more than 790,000 procedures performed annually in the United States [9]. Given this growing burden, there is strong clinical and public health interest in understanding which patients progress to surgery, how early interventions might prevent surgical need, and how pre-surgical factors influence post-surgical outcomes. There is a lack of longitudinal analysis to study how patients progress to TKR, with much orthopedic surgery research focusing on cross-sectional predictors or post-surgical recovery, using measures such as self-reported pain [10], quality of life index EQ-5D [11, 12], enhanced recovery after surgery (ERAS) based outcomes [13]. As a result, little is known about how patients progress through diagnoses and care events over time prior to TKR, or whether distinct pre-surgical subtypes follow systematically different trajectories. Identifying such subtypes is clinically relevant for anticipating disease progression, improving prehabilitation, guiding surgical timing, and addressing disparities in access to care.

At the same time, the diagnostic and clinical trajectories leading up to TKR vary substantially across patients [1]. These multi-year trajectories reflect a combination of chronic and recurring MSD or MSD-related conditions (e.g., osteoarthritis, joint pain, obesity), acute events (e.g., falls, fractures), and variable management such as physical therapy, pain (e.g., opioid use), and weight management. Patients’ diagnostic and clinical trajectories prior to TKR are highly heterogeneous, potentially reflecting substantial variation in symptom progression, comorbidity, and especially care-seeking patterns [2, 3]. Prior studies have shown that pre-surgical comorbidity/diagnosis, socioeconomic deprivation, and psychological factors such as anxiety and depression strongly influence surgical outcomes, yet the longitudinal structure of pre-surgical care pathways (i.e., trajectories of surgery-related diagnosis/event) that give rise to these differences remain poorly characterized [4]. Most existing TKR research focuses on cross-sectional risk factors or post-surgical outcomes, leaving a gap in understanding how patients progress through diagnostic events over time [5, 10]. Trajectory-level analysis is therefore essential for identifying subgroups with distinct care-seeking patterns, informing more personalized or timely interventions prior to surgery. Understanding pre-surgical care-seeking trajectories requires analytic methods that can represent the ordering, timing, and recurrence of diagnosis events over multiple years. However, standard clustering algorithms (e.g., K-means, Gaussian Mixture Models) are not appropriate for longitudinal electronic health records (EHR) trajectories because they assume fixed-length numeric vectors and cannot capture temporal ordering or sparse, irregular events [14, 15]. Classical sequence clustering methods such as unweighted edit distance or Dynamic Time Warping (DTW) capture temporal ordering or alignment but treat diagnoses as unrelated symbols [16, 17]. In musculoskeletal care, diagnoses such as joint pain, osteoarthritis, obesity, and soft-tissue disorders often appear together across patient trajectories, reflecting how individuals repeatedly enter and re-enter the healthcare system for related symptoms rather than isolated disease events. Ignoring these semantic relationships can therefore obscure meaningful similarities between patient trajectories.

In parallel, substantial methodological work in biomedical informatics demonstrates that longitudinal EHR trajectories contain meaningful structures that can be uncovered through sequence analysis. A growing body of research has applied sequence similarity metrics, clustering, and pattern-mining approaches to characterize clinical pathways across a range of conditions. For example, several studies have mined diagnostic histories using pairwise sequence similarity and clustering to identify heterogeneous care trajectories in large EHR datasets [18]. Other work has focused on surgical populations, such as the discovery of care pathways among patients undergoing pulmonary resection for oncologic indications using temporal similarity metrics [19]. Pattern-mining algorithms have also been used to uncover clinical sequences associated with progression or adverse outcomes in congestive heart failure, revealing frequently recurring diagnostic and treatment motifs [20]. Additional studies have applied sequence-alignment–based methods to cluster patients with rheumatoid arthritis, leveraging the temporal ordering of therapies, medications, and appointments to reveal clinically relevant subgroups [21]. Collectively, these approaches highlight the value of trajectory-based analytics for uncovering heterogeneous care patterns across chronic and acute conditions. However, their application to orthopedic surgery, and particularly to the multi-year diagnostic and care-seeking sequences that precede TKR, remains limited. Moreover, most existing methods do not account for essential features of musculoskeletal disease trajectories, including variable timing between events, recurrence patterns, and the semantic similarity of diagnoses.

Taken together, prior studies and methodologies demonstrate the utility of sequence-based methods for analyzing longitudinal EHR data, but they have largely focused on other clinical domains, relied on simplified representations of time, or treated diagnosis events as interchangeable symbols without considering clinical meaning. These gaps motivate the need for a time-aware and uncertainty-aware framework capable of characterizing heterogeneous longitudinal EHR trajectories based on the ordering, recurrence, timing, and semantic categories of clinical events. The objective of this study was to develop and evaluate the time-aware sequence clustering (TASC) framework for longitudinal EHR trajectory analysis and to demonstrate its application using pre-surgical care-seeking pathways preceding TKR. We further incorporated probability-based cluster membership analyses to quantify confidence and identifiability of trajectory subtypes from partial longitudinal records. As a proof-of-concept application, we examined whether the resulting trajectory clusters exhibited distinct sociodemographic characteristics, diagnostic/comorbidity patterns, time-to-surgery distributions, opioid utilization, and post-surgical quality-of-life outcomes. Together, these analyses demonstrate the ability of TASC to identify stable and interpretable trajectory structures from heterogeneous longitudinal EHR data and provide a foundation for future applications in longitudinal clinical sequence analysis.

## Methods

### Study design and data resource

We used the *All of Us* Research Program Dataset v8 (Registered and Controlled Tier), including EHR and survey responses from participants who were 18 years or older and living in the United States from 05/06/2018 to 10/01/2023 [22]. We included patients in the study if they agreed to share EHR and survey data. We identified 2,178 patients who underwent TKR, and for each patient we selected the first occurrence of TKR as the index event. We extracted all diagnosis records prior to the index date, with timestamps, concept identifiers, and visit information. Diagnoses unrelated to MSD or relevant comorbidities were removed as part of preprocessing. For each patient, the analytic window included all diagnoses from the earliest MSD-related event up to the TKR date.

### Time-aware sequence clustering framework overview

**Fig. 1:**
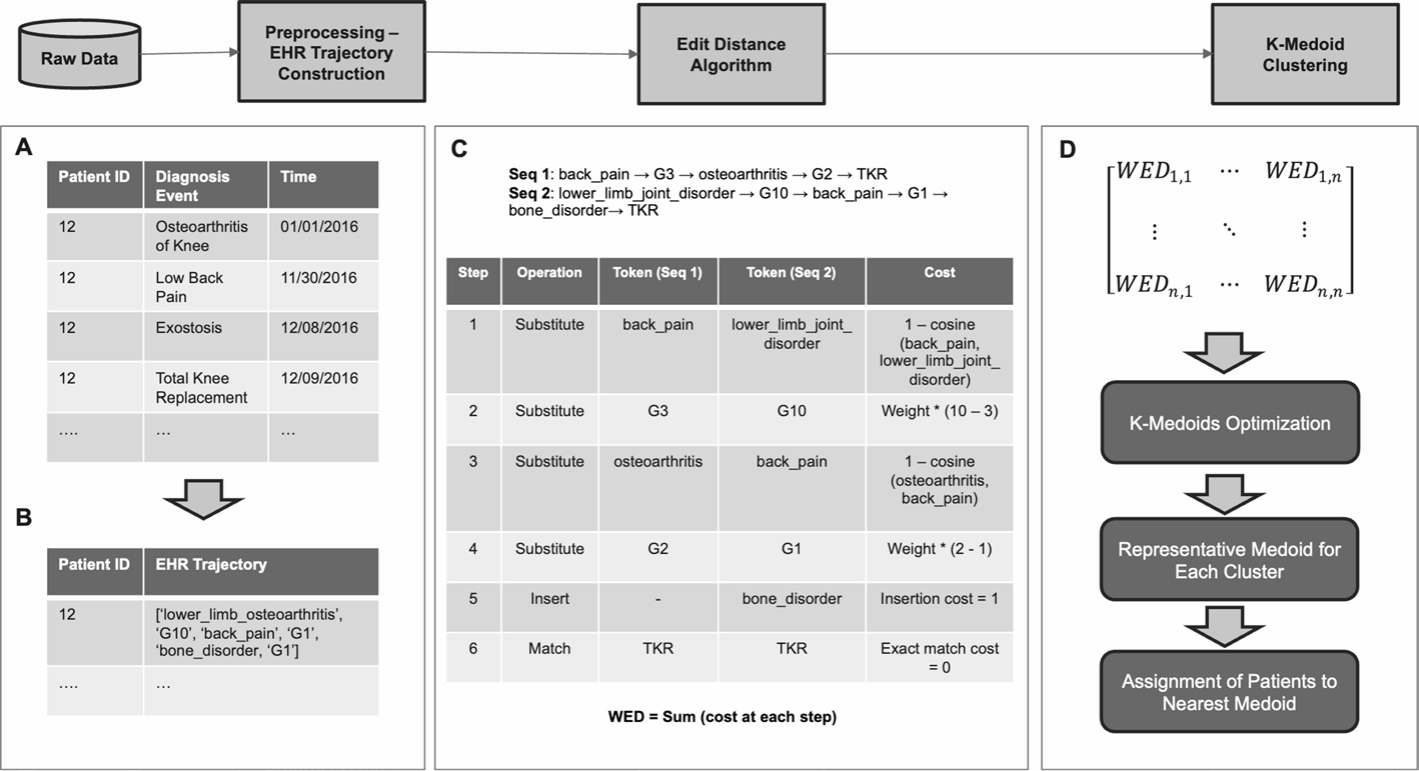
Time-Aware Sequence Clustering (TASC) Framework. The TASC framework transforms longitudinal electronic health record (EHR) data into time-aware care-seeking trajectories and clusters patients based on sequence similarity. (**A**) Raw EHR data consist of patient-level musculoskeletal diagnosis events with associated timestamps. (**B**) Events are converted into ordered diagnosis–time sequences by inserting discretized time-gap tokens (G1–G10) between consecutive diagnosis tokens, where gap tokens represent deciles of the empirical distribution of inter-event intervals. (**C**) Pairwise similarity between patient trajectories is computed using a weighted edit distance algorithm that allows insertion, deletion, and substitution operations. Substitution costs are token-specific: diagnosis–diagnosis substitutions are weighted by cosine distance between Word2Vec embeddings, time-gap substitutions reflect absolute differences between gap deciles, and insertions or deletions incur a fixed unit cost. A worked alignment example illustrates how individual edit operations contribute to the total distance. (**D**) The resulting weighted edit distance matrix is used as input to k-medoid clustering, yielding representative medoid trajectories for each subtype and assignment of patients to the nearest medoid based on minimal sequence distance

We developed the TASC framework to characterize heterogeneous diagnostic and care-seeking trajectories preceding total knee replacement (Fig. 1). The framework transforms each patient’s longitudinal EHR history into a temporally ordered sequence of clinically grouped diagnosis events, interleaved with time-gap tokens that encode the interval between encounters. We then compute pairwise similarities between trajectories using a custom weighted edit distance that accounts for semantic similarity between diagnoses and variation in timing between events. The resulting distance matrix serves as input to a clustering algorithm, enabling the identification of patient subgroups with distinct pre-surgical pathways. This framework integrates clinical meaning, temporal structure, and trajectory irregularity; features essential for understanding the complexity of MSD care progression prior to surgery.

The design of TASC is informed by prior work in longitudinal sequence analysis and clinical pathway modeling, which has shown that distance–based methods are well suited for comparing variable-length, irregular healthcare trajectories [18, 19, 21]. Sequence representations preserve the ordering and recurrence of diagnostic events, while explicit encoding of time gaps captures clinically meaningful variation in care intensity and spacing that is often lost in event-only models. Incorporating semantic similarity between diagnoses further reflects the reality that many musculoskeletal conditions represent related manifestations within care-seeking pathways rather than independent disease events.

As a baseline, we applied standard edit-distance clustering to trajectories of diagnoses. This baseline reflects a naive sequence-similarity approach that does not incorporate temporal spacing or semantic similarity between events.

### Preprocessing - EHR trajectory construction

We represented each patient’s pre-surgical care history as a temporally ordered sequence of tokens. Tokens consisted of two types:

- Event tokens, corresponding to MSD-related diagnosis categories (e.g., lower_limb_osteoarthritis, soft_tissue_disorder) and relevant comorbidities (e.g., falls, fractures, obesity, vitamin D deficiency).
- Time-gap tokens (G1–G10), representing discretized intervals between consecutive clinical events.

Event and time-gap tokens were interleaved to preserve both the ordering of diagnoses and the temporal spacing between them (e.g., event token → time-gap token → event token).

These events potentially capture the episodic, irregular nature of MSD progression and more importantly can reflect how TKR patients enter and re-enter care over time. Diagnosis codes were grouped into clinically meaningful categories to generate event tokens (see details in Supplementary Materials). In this study, we anchor trajectories at each patient’s earliest MSD-related diagnosis recorded in the EHR at which musculoskeletal care-seeking behavior becomes observable in routine clinical data. This design enables characterization of upstream musculoskeletal burden, comorbidities, and care utilization patterns that may precede eventual TKR.

Time gaps between consecutive events were computed in days. To account for heterogeneous timing, each inter-event time gap was binned into deciles (e.g., G1, G2, …, G10), representing short to long intervals (see Supplementary Fig. 1 for details). Because chronic MSD-related diagnoses (e.g., low back pain, osteoarthritis) are repeatedly re-recorded across visits without representing new clinical information, we retained only the first and last occurrence of each event token. This reduces redundancy driven by encounter-level documentation and prevents the edit distance from being dominated by sequence length rather than clinically meaningful transitions. Patients with fewer than two qualifying events were excluded from the analysis. Keeping the onset and final occurrence preserves the temporal span of each condition while minimizing noise from repeated record entries.

### Edit distance algorithm

**Fig. 2:**
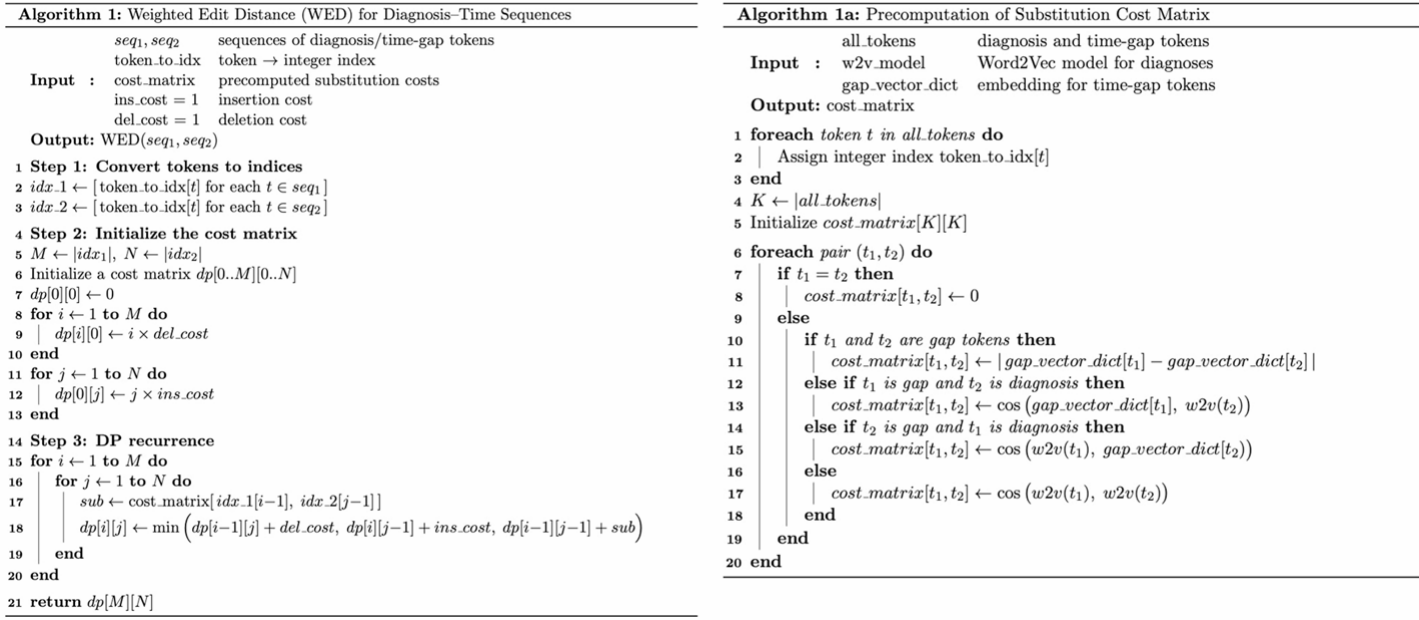
Weighted edit distance (WED) Algorithm

We define a weighted edit distance, $\:\mathrm{W}\mathrm{E}\mathrm{D}(se{q}_{1},se{q}_{2})$. This was a pairwise distance measure that quantifies the minimum cost of transforming one diagnosis–time sequence into another using deletion, insertion, and substitution operations (Fig. 2).

- Insertion: adding a token to a sequence.
- Deletion: removing a token from a sequence.
- Substitution: replacing one token with another.

Each edit operation incurs a cost, which quantifies the dissimilarity introduced by that operation. Insertion and deletion operations were assigned a fixed unit cost. Substitution costs were token-specific and depended on the semantic similarity between tokens.

Here, $\:se{q}_{1}$ and $\:se{q}_{2}$ denote two patient-specific longitudinal trajectories, each represented as an ordered sequence of discretized tokens. Each token is either a diagnosis event or a time-gap token G1 through G10, where gap tokens represent deciles of the empirical distribution of inter-event intervals. Time-gap tokens were encoded as scalar values in a shared embedding space, so that the substitution cost between two gaps is the absolute difference between their decile values. Deletion and insertion each have a constant cost of 1. Substitution costs are determined from a precomputed token–token cost matrix: diagnosis–diagnosis pairs use the cosine distance between their Word2Vec embeddings, time–time pairs use the absolute difference between their gap encodings, and diagnosis–time pairs use the cosine distance between the diagnosis embedding and the gap embedding (Fig. 2).

To incorporate clinical semantic similarity among tokens, we trained a Word2Vec model on all diagnosis sequences and computed cosine similarity between diagnosis-token embeddings [23]. Accordingly, the substitution cost $\:{c}_{sub}$between two diagnosis tokens was defined using Eq. 1 where $\:e\left(a\right)$ and $\:e\left(b\right)$ denote the embedding vectors of diagnosis tokens *\:a* and *\:b*, respectively.

1 $$\>{c_{{\rm{sub}}}}(a,b) = 1 - {\rm{cos}}(e(a),e(b))$$

This formulation allowed clinically similar diagnoses (e.g., “lower_limb_joint_disorder” and “osteoarthritis”) to incur lower substitution penalties. The resulting weighted edit distance matrix quantified pairwise dissimilarity between all patient trajectories.

### Clustering and stability analysis

Patient trajectories were clustered using K-Medoids, with the precomputed edit-distance matrix as input [24]. The optimal number of clusters was selected using the silhouette score. To assess the robustness of the clustering solution, we conducted a subsampling-based stability analysis following principles recommended in Hennig’s framework for cluster stability [25]. We first generated a reference clustering by applying our full trajectory-clustering pipeline to the entire cohort. We then performed 300 perturbation iterations in which 10% of patients were randomly removed, the distance matrix was recomputed for the remaining patients, and clustering was repeated using the same number of clusters. Because cluster labels can permute across runs, cluster assignments from each perturbed iteration were aligned to the reference solution using maximum-overlap matching (Hungarian algorithm) [26]. Stability was evaluated using the adjusted Rand index (ARI) computed on the overlapping set of patients for each iteration to measure the similarity between the two clustering [27]. The distribution of ARI values was used to evaluate the sensitivity of the clustering structure to modest perturbations of the data.

### Statistical analysis and cluster interpretation

We evaluated cluster differences across demographic, socioeconomic, and clinical characteristics extracted from *All of Us* EHR and surveys responses. We selected variables that are routinely available in *All of Us*, clinically interpretable, and commonly used to contextualize differences in healthcare utilization, social support, and patient-centered recovery following orthopedic surgery.

- Demographics: age at surgery, sex at birth (female vs. male).
- Socioeconomics: insurance type, living alone (vs. with others).
- Time-to-surgery: days between earliest MSD diagnosis and TKR.
- Quality of life: EQ-5D index score, derived from mobility, self-care (dressing and washing), usual activities, pain, and emotional health domains from self-reported survey responses [6, 7].
- Diagnosis/comorbidity burden: occurrence of MSD-related diagnoses and comorbidities.
- Opioid utilization: pre- and post-surgical chronic opioid use, defined according to [28–30].

**Fig. 3:**
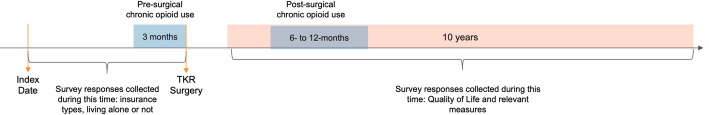
Timeline for variable selection from EHR and survey

Survey-based sociodemographic characteristics, including insurance type and living situation (living alone vs. not), were from responses collected at any available time point prior to surgery within the observation period following each patient’s index date. These variables were used to characterize pre-surgical social context across trajectory subtypes. Quality-of-life measures were obtained from survey responses collected during the 10-year period following surgery, after applying a 3-month washout period to avoid capturing acute post-surgical effects (Fig. 3). There was at least one survey response related to quality-of-life measures and living alone or not during the corresponding time window for all included patients. Accordingly, analyses were performed using available responses within this predefined observation windows without imputation. Responses closest to the surgery time were selected. For missing responses of insurance types, we labeled them as none or unknown.

For statistical analyses, differences across clusters were evaluated using one-way ANOVA for continuous variables and chi-square tests for categorical variables. Time-to-surgery was evaluated using Kaplan-Meier curves. P-values were adjusted for multiple comparisons using the Benjamini-Hochberg procedure, with *p* < 0.05 indicating significance [31].

### Pattern mining

To complement sequence clustering, we applied frequent-pattern mining to identify common subsequences and recurring diagnostic motifs within clusters. We used the Apriori algorithm to extract frequent itemset because it provides an interpretable, rule-based representation of co-occurring diagnoses and is well suited for identifying stable combinations of clinical events that appear across patients with heterogeneous trajectories [32]. Unlike the weighted edit-distance clustering, which focuses on global trajectory similarity and temporal alignment, Apriori captures local patterns that may recur repeatedly within clusters but do not dominate the overall sequence structure. This allowed us to detect clinically meaningful diagnostic motifs, such as common co-occurrence of MSD-related conditions or transitions that occur early versus late in the pre-surgical pathway. Frequent-pattern mining thus served as a secondary validation step and enhanced cluster characterization by highlighting patterns not fully captured through distance-based trajectory analysis. Detailed results from this analysis are provided in the Supplementary Materials.

### Probability-based characterization of cluster membership and uncertainty

To quantify uncertainty in cluster membership and assess how pathway identifiability evolves as clinical information accrues, we performed a probability-based cluster membership analysis using both full and partial diagnosis sequences. For each patient, the full pre-surgical trajectory (from the earliest MSD-related diagnosis to TKR) served as a reference, and additional analyses were conducted using partial trajectories defined relative to the first documented knee osteoarthritis (OA) encounter. Two types of partial trajectories include pre-knee-OA (from the earliest MSD-related diagnosis to first knee OA) and post-knee-OA (from first knee OA to TKR).

Let *\:x* denote a patient’s diagnosis sequence (full or partial) and $\:{m}_{k}$ denote the medoid sequence of cluster $\:k\in\:\{1,\dots\:,K\}$ obtained from full-trajectory clustering. We computed the weighted edit distance $\:{d}_{k}\left(x\right)$ between *\:x* and each medoid $\:{m}_{k}$ using the time-aware edit-distance metric described above. Distances were transformed into soft cluster membership probabilities using a SoftMax function:

2 $$p(z = k\mid x){\rm{ = }}{{{\rm{exp}}\left( {{\rm{ - }}{{\rm{d}}_{\rm{k}}}\left( {\rm{x}} \right){\rm{/}}\tau } \right)} \over {\sum\limits_{{\rm{j = 1}}}^{\rm{K}} {{\rm{exp}}\left( {{\rm{ - }}{{\rm{d}}_{\rm{j}}}\left( {\rm{x}} \right){\rm{/}}\tau } \right)} }},$$

where $\:\tau\:$ is a temperature parameter controlling the sharpness of the resulting probability distribution.

For full trajectories, these probabilities provide a reference measure of the maximum cluster separability achievable with complete longitudinal information. For partial trajectories, they quantify the relative similarity of incomplete care histories to each cluster prototype. To summarize uncertainty, we defined confidence as the maximum membership probability $\:{\mathrm{m}\mathrm{a}\mathrm{x}}_{k}p(z=k\mid\:x)$, representing the strength of assignment to the most likely subtype. We further assessed concordance as the agreement between the subtype with highest probability based on a partial trajectory and the subtype assigned using the corresponding full trajectory.

Together, these measures allow explicit characterization of cluster membership uncertainty and the extent to which pathway subtypes become distinguishable under incomplete clinical information. This analysis is intended to describe uncertainty and pathway consolidation over time, rather than to support real-time clinical decision-making.

## Results

### TASC framework performance and uncertainty quantification

After excluding patients with unqualified, short trajectories, we used data from 2,052 TKR patients in the clustering analysis. TASC produced five clusters with moderate separation. Our baseline clustering yielded only two groups, which primarily differentiated patients by overall sequence length (i.e., number of recorded diagnosis events) rather than clinically meaningful pathway patterns. This demonstrates that naive edit-distance metrics collapse the structure of MSD care trajectories into a trivial partition, motivating the need for a distance function that incorporates both temporal and semantic information.

**Fig. 4:**
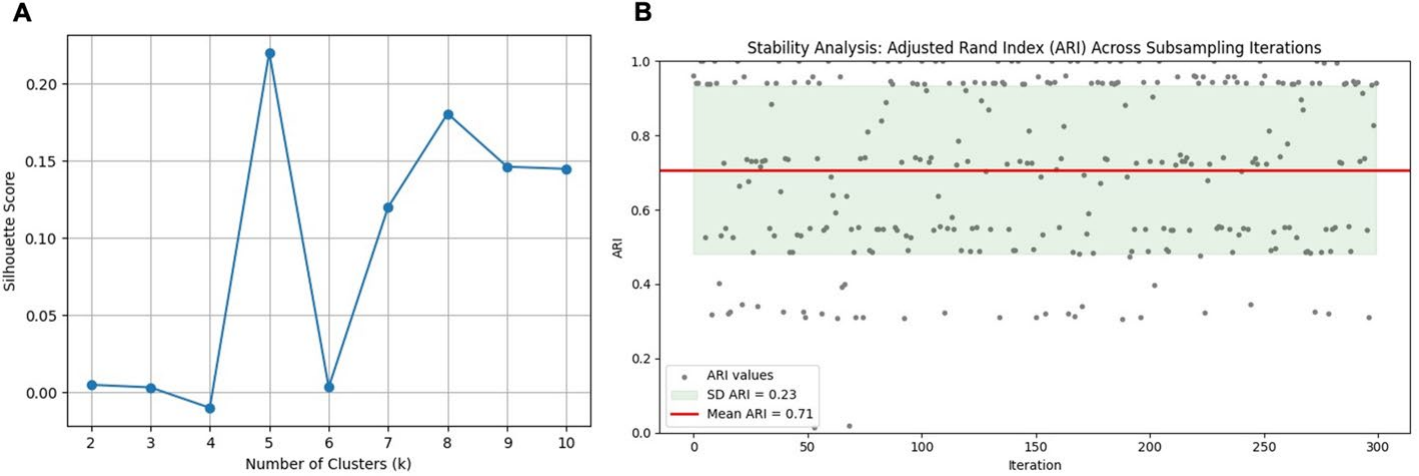
Cluster evaluation. (**A**) Silhouette scores across candidate values of k, with a peak at k = 5 indicating optimal cluster separation; (**B**) Subsampling-based stability analysis showing the distribution of ARI values across 300 perturbation iterations; higher ARI values reflect greater consistency with the reference clustering

In contrast, the proposed distance metric, which incorporates both semantic substitution costs and time-gap weighting, produced a five-cluster solution. The silhouette curve across candidate values of *\:k* (Fig. 4A) showed a clear peak at *\:k=5* (silhouette = 0.22), indicating that five clusters provided the best separation in the weighted edit-distance space. Although the silhouette magnitude is modest, as expected for high-dimensional, irregular EHR sequences, it reflects meaningful structure relative to alternative values of *\:k*, with surrounding solutions exhibiting notably poorer separation.

To evaluate the robustness of the five-cluster solution, we conducted a subsampling-based stability analysis. Across 300 perturbation iterations, each involving random removal of 10% of the cohort and re-clustering, the adjusted Rand index (ARI) between each perturbed solution and the reference clustering had a mean of 0.71 and a standard deviation of 0.23 (Fig. 4B). This distribution demonstrates good stability: most iterations yielded ARI values well above 0.6, indicating that the core cluster structure is preserved under modest perturbations, while variability among lower-ARI iterations likely reflects borderline or transitional trajectories. The stability band (mean ± SD) further highlights the consistency of cluster boundaries across repeated subsamples.

For the frequent-pattern mining, no recurring diagnostic patterns were identified in Cluster 0, consistent with its sparse diagnostic histories. In contrast, Cluster 1 exhibited joint-disorder and obesity–osteoarthritis groupings, Cluster 2 showed enriched combinations linking enthesopathies with osteoarthritis, and Cluster 3 displayed soft-tissue and enthesopathy motifs. Cluster 4 contained the broadest and most complex co-occurrence sets, spanning osteoarthritis, spondylosis, muscle and bone disorders, and back pain. These findings support the trajectory-based cluster structure by showing certain diagnostic combinations that consistently appear within the same subtypes. More details can be found in the Supplementary Table 1.

**Table 1:** Concordance and confidence of full and partial trajectory analysis by TASC-derived clusters

Cluster	Full trajectory		Pre-first-knee-OA		Post-first-knee-OA	
	Concordance	Median Confidence (% confidence > = 0.7)	Concordance	Median Confidence (% confidence > = 0.7)	Concordance	Median Confidence (% confidence > = 0.7)
0 (*n* = 109) Fast progression to surgery	1	0.979 (70.4%)	1	0.870 (66.1%)	1	0.933 (90.8%)
1 (*n* = 157) Joint pain, obesity	0.711	0.688 (23.8%)	0.573	0.485 (0%)	0.013	0.894 (73.9%)
2 (*n* = 97) Joint pain, fracture	0.444	0.559 (13.3%)	0.351	0.481 (0%)	0.031	0.890 (75.3%)
3 (*n* = 479) Soft-tissue dominant	0.973	0.989 (90.8%)	0.338	0.501 (20.3%)	0.296	0.888 (73.3%)
4 (*n* = 594) Complex, chronic	0.947	0.999 (92.2%)	0.093	0.586 (41.2%)	0.337	0.905 (78.3%)
Overall (*n* = 1436)	0.907	0.985	0.313	0.521	0.318	0.898

Probability-based analyses further demonstrated how TASC quantifies confidence and identifiability of trajectory assignment under incomplete longitudinal information. Integrating full-trajectory clustering with partial-pattern analyses allowed us to examine how and when care-seeking pathway subtypes emerge as distinguishable patterns over the course of routine clinical care. Among the 2,052 patients used for analysis, 1,436 had a documented knee OA diagnosis (Table 1). A fast progression subtype (Cluster 0) was highly distinctive, showing near-perfect concordance and high confidence both before and after knee OA documentation, consistent with younger age, minimal comorbidity, and rapid progression to surgery. Obesity- (Cluster 1) and fracture-associated (Cluster 2) pathways showed higher concordance with the final subtype prior to knee OA documentation, suggesting that early MSD history may capture risk factors relevant to subsequent care trajectories. In contrast, soft-tissue–dominant (Cluster 3) and complex chronic (Cluster 4) pathways exhibited low concordance even after knee OA recognition, indicating that these trajectories are inherently diffuse and require longitudinal accumulation of events to be meaningfully characterized.

### Representative longitudinal care-utilization patterns identified by TASC

To illustrate the interpretability of the TASC framework, we summarized the representative longitudinal care-utilization patterns associated with each cluster using K-medoid trajectories and descriptive trajectory characteristics. Representative trajectory (i.e., cluster centroid) for each subtype can be found in Table 2.

- Cluster 0 (“fast progression to surgery”) comprised younger patients with few MSD-related precursor events, limited visit frequency, a higher proportion of private insurance, and favorable post-surgical quality-of-life scores.
- Cluster 1 (“joint pain, obesity”) demonstrated a relatively short progression window, lower female representation, greater reliance on public insurance, and the highest post-surgical quality-of-life outcomes.
- Cluster 2 (“joint pain, fracture”) included younger patients with private insurance and self-reported emotional problems (Supplementary Table 2), containing a mixture of joint pain and fracture-related events.
- Cluster 3 (“soft-tissue dominant”) showed prolonged trajectories characterized by soft-tissue diagnoses, extended conservative management such as higher rate of chronic opioid use.
- Cluster 4 (“complex, chronic”) represented the oldest and most clinically burdened group, with high prevalence of MSD-related precursor events, greater reliance on public insurance, higher likelihood of living alone, chronic opioid use, and the lowest post-surgical quality-of-life scores.

**Table 2:** K-Medoid Centroid / Representative Trajectory by TASC-derived Cluster

Cluster	Representative Trajectory
0 (*N* = 310) Fast progression to surgery	lower_limb_osteoarthritis → G5 → joint_disorder → G1 → TKR
1 (*N* = 210) Joint pain, obesity	soft_tissue_disorder → G2 → osteoarthritis → G1 → lower_limb_osteoarthritis → G2 → obesity → G1 → obesity → G1 → TKR
2 (*N* = 258) Joint pain, fracture	osteoarthritis → G1 → hip_fracture → G2 → back_pain → G4 → neck_disorder → G1 → enthesopathies → G1 → hip_fracture → G2 → joint_disorder → G1 → TKR
3 (*N* = 606) Soft-tissue dominant	back_pain → G6 → joint_disorder → G5 → soft_tissue_disorder → G4 → enthesopathies → G2 → enthesopathies → G4 → lower_limb_osteoarthritis → G1 → osteoarthritis → G4 → soft_tissue_disorder → G3 → joint_disorder → G3 → TKR
4 (*N* = 668) Complex, chronic	obesity → G6 → joint_disorder → G5 → lower_limb_osteoarthritis → G3 → back_pain → G2 → lower_limb_joint_disorder → G2 → soft_tissue_disorder → G6 → obesity → G5 → neck_disorder → G5 → enthesopathies → G6 → enthesopathies → G4 → lower_limb_joint_disorder → G1 → soft_tissue_disorder → G4 → back_pain → G4 → osteoarthritis → G4 → TKR

* Diagnosis tokens represent grouped musculoskeletal or related clinical events derived from ICD-9/10 codes (see Supplementary Materials “Diagnostic event grouping and distribution” for details). Time-gap tokens (G1–G10) denote discretized intervals (i.e., 10 deciles) between consecutive diagnosis events, where higher values correspond to longer time between events. Repeated diagnosis tokens indicate recurring clinical encounters or reappearances of the same condition over time, rather than duplicate coding

Each trajectory represents the K-medoids cluster medoid, defined as the observed patient trajectory with minimal average distance to all other trajectories in the cluster. Trajectories are shown as ordered sequences of diagnosis tokens interleaved with discretized time-gap tokens

### Associations with sociodemographic and post-surgical outcomes

We further examined whether TASC-derived trajectory structures were associated with distinct sociodemographic and post-surgical outcome patterns. Across the five trajectory-defined subtypes, we observed clear differences in sociodemographic characteristics (Table 3). Patients in Cluster 0 (“fast progression to surgery”) were relatively young on average (53.6 years) and had high quality-of-life scores, consistent with relatively low comorbidity burden and earlier surgical referral. Clusters 3 and 4 contained older patients (mean age at surgery of 60.0 and 62.7 years, respectively), suggesting more prolonged or complex disease trajectories. The sex distribution varied substantially, with Cluster 4 showing a predominance of women (74%), in line with known sex differences in chronic pain and musculoskeletal fragility. Insurance types also differed across clusters: public insurance coverage was most common in the obesity-dominant Cluster 1 (63%) and complex Cluster 4 (64%), whereas Cluster 0 had a higher proportion of private insurance (33%). Living alone was most prevalent in Cluster 4 (31%), which may reflect greater functional limitations or social vulnerability.

**Table 3:** Patient Socio-demographics by TASC-derived Clusters

Cluster	Age*** Years Mean (SD)	Sex*** Female Count (proportion)	QoL EQ-5D Index*** Mean (SD)	Insurance Type**** Count (proportion)			Living Alone**** Count (proportion)	
				Public	Private	None or Unknown	Yes	No
0 (*N* = 310) Fast progression to surgery	53.6 (16.5)	197(0.64)	0.58 (0.28)	155 (0.50)	103 (0.33)	52 (0.17)	56 (0.18)	254 (0.82)
1 (*N* = 210) Joint pain, obesity	58.8 (12.7)	116 (0.55)	0.60 (0.27)	133 (0.63)	55 (0.26)	22 (0.11)	57 (0.27)	153 (0.73)
2 (*N* = 258) Joint pain, fracture	51.7 (15.9)	141 (0.55)	0.54 (0.30)	103 (0.40)	80 (0.31)	75 (0.29)	44 (0.17)	214 (0.83)
3 (*N* = 606) Soft-tissue dominant	60.0 (11.3)	358 (0.59)	0.55 (0.28)	364 (0.60)	129 (0.22)	113 (0.18)	139 (0.23)	467 (0.77)
4 (*N* = 668) Complex, chronic	62.7 (10.0)	495 (0.74)	0.50 (0.30)	428 (0.64)	131 (0.20)	109 (0.16)	207 (0.31)	461 (0.69)

Statistical significance is denoted as *P* < 0.05 (*), *P* < 0.01 (**), *P* < 0.001 (***), and *P* < 0.0001 (****). Abbreviations: standard deviation (SD), quality of life (QoL)

* Values are presented as mean (SD) for continuous variables and count (proportion) for categorical variables. QoL EQ-5D index ranges from 0 (worst) to 1 (best)

**Fig. 5:**
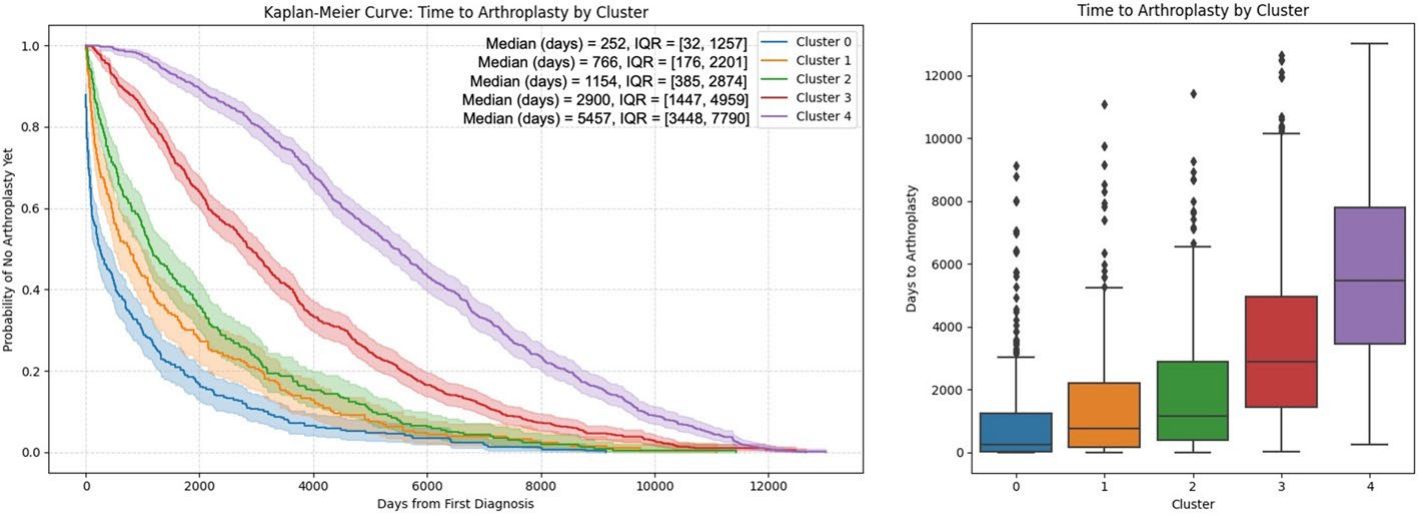
Patient time-to-surgery by clusters. Kaplan–Meier curves (left) and boxplots (right) summarize the distribution of time from the earliest documented musculoskeletal diagnosis to total knee replacement (TKR) across trajectory clusters. Differences in curve shape and median time-to-surgery reflect heterogeneity in pre-surgical care pathways, with some subtypes progressing rapidly to surgery and others exhibiting prolonged care trajectories prior to arthroplasty. The wide range of observed durations (extending beyond 10,000 days) reflects heterogeneity in when patients entered the EHR system and the length of available longitudinal records across individuals, rather than continuous follow-up or missing data over a fixed multi-decade period. Each patient’s trajectory includes all MSD-related diagnoses documented during their observed care period prior to TKR

Time from the first MSD-related event to TKR varied widely across clusters, reflecting distinct patterns of care-seeking behavior (Fig. 5). Cluster 0 exhibited the shortest time to surgery (median = 252 days), consistent with rapid escalation from initial diagnosis to surgical intervention. In contrast, Cluster 4 (“complex, chronic”) demonstrated the longest and broadest time-to-surgery distribution (median = 5457 days), reflecting long-standing multimorbidity, recurrent fragility events, and a protracted course of diagnostic accumulation before surgery.

**Fig. 6:**
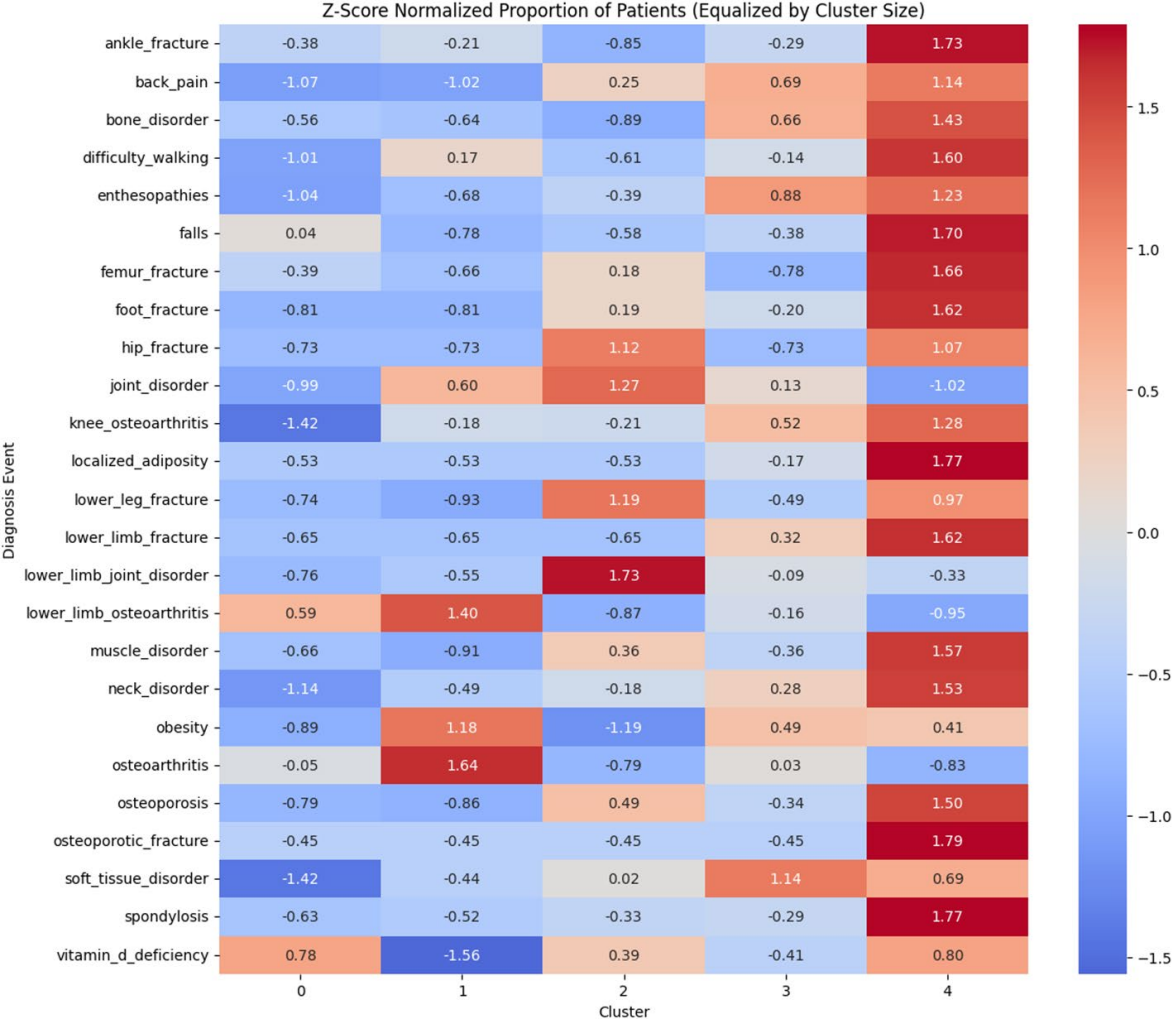
Relative enrichment of diagnosis categories across TASC-derived trajectory subtypes. For each diagnosis category *\:d* and cluster *\:c*, we computed the proportion, Because clusters vary in size, proportions rather than raw counts were used to enable fair comparison across clusters. For each diagnosis, these proportions were then standardized across clusters using z-score normalization: Z_(c,d)_=(p_(c,d)_-μ_d_)/σ_d_ , where μ_d_ and σ_d_ denote the mean and standard deviation of p_(c,d)_ across clusters. Positive z-scores (red) indicate diagnoses that are disproportionately common within a given cluster relative to the cohort average, whereas negative z-scores (blue) indicate diagnoses that occur less frequently than expected. Values near zero (white) reflect average prevalence across clusters

The five clusters differed largely in the types and combinations of MSD-related diagnoses that characterized their pre-surgical histories (Fig. 6). Proportions of diagnosis events were z-score normalized and adjusted for cluster size to allow direct comparison of diagnostic prevalence across clusters. Cluster 0 had a low comorbidity burden and a relatively straightforward progression. Cluster 1 was defined by lower limb osteoarthritis, joint disorders, and high rates of obesity. Cluster 2 combined joint pain with leg or hip fractures, suggesting a fragility-associated pathway. Cluster 3 was characterized by soft-tissue disorders, enthesopathies, and back pain. In contrast, Cluster 4 exhibited the most complex diagnostic profile, with high prevalence of fractures, falls, difficulty walking, spondylosis, muscle and bone disorders, and localized adiposity. This cluster reflects substantial musculoskeletal burden, multimorbidity, and likely frailty.

Patterns of chronic opioid use before and after surgery further differentiated the five subtypes (Table 4). Pre-surgical chronic opioid use was common across all clusters (57–68%), but highest in the two with the longest trajectory and progression: Cluster 3 (66%) and Cluster 4 (68%). Post-surgical chronic opioid use decreased across clusters, but significant difference among clusters remained. Cluster 0 showed the lowest post-surgical chronic opioid use (25%), consistent with its lower comorbidity burden and shorter disease course. In contrast, post-surgical chronic use was highest in Cluster 4 (54%) and Cluster 3 (47%), reflecting their more severe and prolonged musculoskeletal conditions. These patterns suggest that clinical trajectory complexity, fragility, and multimorbidity are closely tied to both pre- and post-surgical opioid requirements, demonstrating important implications for pain management and prehabilitation strategies.

**Table 4:** Pre-surgical and post-surgical chronic opioid use by TASC-derived clusters

Cluster	Pre-surgical Chronic Opioid Use**** Count (proportion)		Post-surgical Chronic Opioid Use**** Count (proportion)	
	Yes	No	Yes	No
0 (*N* = 310) Fast progression to surgery	195 (0.63)	115 (0.37)	77 (0.25)	233 (0.75)
1 (*N* = 210) Joint pain, obesity	128 (0.61)	82 (0.39)	63 (0.30)	147 (0.70)
2 (*N* = 258) Joint pain, fracture	147 (0.57)	111 (0.43)	95 (0.37)	163 (0.63)
3 (*N* = 606) Soft-tissue dominant	400 (0.66)	206 (0.34)	284 (0.47)	321 (0.53)
4 (*N* = 668) Complex, chronic	454 (0.68)	214 (0.32)	361 (0.54)	307 (0.46)

Statistical significance is denoted as *P* < 0.05 (*), *P* < 0.01 (**), *P* < 0.001 (***), and *P* < 0.0001 (****)

## Discussion

The five trajectory subtypes identified by TASC demonstrate heterogeneous longitudinal care-utilization patterns and illustrate how temporal EHR trajectories can capture differences in diagnostic complexity, healthcare utilization, and progression to surgery across patient groups. Patients in Cluster 0 exhibited rapid progression to surgery with minimal diagnostic complexity, suggesting relatively straightforward clinical trajectories and fewer barriers to surgical treatment, potentially reflecting better healthcare access, clearer symptom profiles, or greater readiness to pursue intervention. In Cluster 1, despite higher rates of public insurance and living alone, patients achieved the highest post-surgical quality-of-life scores, indicating that timely surgical intervention may effectively address unmet needs in this pain- and obesity-associated phenotype. Cluster 2 combined joint pain with fracture-related events and elevated emotional health concerns, highlighting the potential value of more integrated physical and psychological care models for individuals whose MSD pathways are shaped by both degeneration and acute injury [33]. The prolonged trajectories and high chronic opioid use observed in Cluster 3 suggest potential extended conservative management, possibly driven by the complexity of soft-tissue–dominant MSD conditions [34]. Finally, Cluster 4 reflects a multimorbid, socially vulnerable population with substantial musculoskeletal burden, reliance on public insurance, and late surgical intervention, raising the possibility that cumulative clinical, social, and economic barriers may delay treatment and contribute to poorer post-surgical outcomes. Together, these findings underscore the heterogeneity of pre-surgical pathways and highlight opportunities to tailor care strategies to the needs of distinct patient subgroups. Clinically, these findings suggest that not all musculoskeletal care pathways are equally identifiable early in the disease course. For some patients, early MSD encounters already resemble eventual care patterns, supporting early non-surgical interventions such as weight and physical activity management. For others, particularly those with diffuse soft-tissue involvement or multimorbidity, pathway identity consolidates only after prolonged care, underscoring the limitations of early categorization. Together, these findings illustrate the ability of TASC to capture heterogeneous pre-surgical care trajectories and provide interpretable population-level context for understanding variation in musculoskeletal care utilization prior to TKR.

Importantly, the identified trajectory clusters should be interpreted as data-driven longitudinal care-utilization patterns rather than validated clinical phenotypes or disease progression stages. Because trajectories were anchored at the earliest MSD-related documentation rather than a standardized knee-specific disease onset, baseline heterogeneity in disease stage may influence pathway structure and interpretation relative to knee OA progression. In addition, ICD-based trajectory patterns may partially reflect documentation practices, coding variability, and healthcare utilization behaviors rather than distinct biological entities, particularly among overlapping musculoskeletal diagnoses with nonspecific clinical presentations. While the probability-based analyses improve interpretability by quantifying confidence and identifiability of pathway assignments, the identified clusters primarily demonstrate the capabilities of the TASC framework and require additional validation through approaches such as chart review, operational clinical definitions, and external replication before potential clinical deployment.

To our knowledge, this is the first study to apply a time-aware trajectory clustering framework to characterize pre-surgical diagnostic pathways in patients undergoing TKR. By modeling longitudinal event sequences together with analyzing sociodemographic and clinical characteristics, we provide a more comprehensive view of patient progression than prior work focused on cross-sectional risk factors or isolated clinical events. Methodologically, our weighted edit-distance approach explicitly incorporates both clinical semantics and inter-event time gaps, an aspect overlooked by most existing sequence clustering algorithms, allowing us to capture not only what diagnoses occurred but also when and in what temporal structure they accumulated [18]. The resulting subtypes reveal meaningful heterogeneity in care-seeking behavior, pain and comorbidity patterns, and opioid exposure, offering opportunities for earlier identification of patients who may benefit from timely intervention and more personalized care planning such as weight [35], emotion [36], and pain management [37]. By incorporating probability-based cluster membership, TASC provides an interpretable view by quantifying the confidence of subtype identification from diagnostic patterns as trajectories evolve over time. Complementary frequent-pattern mining further reinforced the distinctiveness of these trajectory subtypes by revealing subtype-specific co-occurring diagnostic patterns, providing additional validation of the longitudinal clustering results. These trajectory-based insights also establish a foundation for future hypothesis-driven investigations, including whether cluster-tailored management pathways can improve functional outcomes or reduce persistent opioid use. This work demonstrates the value of integrating temporal dynamics and clinical context to advance precision musculoskeletal and surgical care.

Several limitations and future directions should be noted. First, EHR-derived trajectories depend on encounter patterns and coding practices and thus may incompletely capture symptoms managed outside of healthcare settings or diagnoses that are not routinely documented. Clinic-level heterogeneity was not discussed due to the absence of clinic identifiers in the All of Us. Retaining only the first and last occurrence of repeated diagnoses removes explicit recurrence counts and may collapse multi-year utilization into a single event span. In this framework, chronicity is therefore captured through overall trajectory duration, temporal spacing between events, and diagnostic diversity rather than raw visit frequency, and alternative representations explicitly modeling recurrence intensity may provide complementary insights in future work. Second, while our weighted edit distance incorporates clinical semantics and timing information, the approach still relies on grouped diagnosis concepts and may not fully represent the granularity or variability of underlying clinical presentations. Third, although our stability analysis suggests a robust five-cluster solution, trajectory clustering remains sensitive to choices in preprocessing, event grouping, and distance parameterization. Moreover, associations between cluster membership and post-surgical outcomes are observational and may reflect underlying confounding factors rather than causal relationships. Another key limitation of our experiment design is that anchoring trajectories at the earliest MSD diagnosis does not align all patients relative to knee OA symptom onset or severity. As a result, the identified subtypes should not be interpreted as representing stages of knee OA progression. Instead, they reflect longitudinal patterns of musculoskeletal care-seeking captured in routine EHR data. However, the TASC framework itself is agnostic to the choice of temporal anchor and event definitions. A clinically complementary line of work would anchor trajectories at the first symptomatic knee OA encounter following a washout period and incorporate richer event types such as physical therapy utilization, procedures, imaging, and laboratory data. Such an extension would be well suited to studying biological severity progression and treatment response subtypes in the future. Finally, because diagnosis embeddings were trained on the same trajectories that were subsequently clustered, the resulting substitution costs reflect cohort- and context-specific diagnostic co-occurrence patterns, which may limit external generalizability. Accordingly, the identified clusters should be interpreted as data-driven representations of care-seeking behavior within this healthcare context rather than universally transferable phenotypes, and future work should assess portability using external embeddings or cross-dataset validation. These considerations highlight opportunities for refinement using more granular phenotyping, alternative sequence representations, and external, prospective validation.

## Conclusion

By applying a time-aware sequence clustering framework to EHR-derived musculoskeletal trajectories, we identified five robust and interpretable longitudinal pre-surgical subtypes among patients undergoing total knee replacement. These subtypes differed systematically in sociodemographic context, diagnostic composition, opioid exposure, and timing of surgical progression, reflecting diverse pathways through which patients arrive at surgery. Our findings illustrate the potential of trajectory-based analytics to characterize heterogeneity in MSD progression and to support more personalized, timely, and equity-aware approaches to surgical care. This framework establishes a foundation for future investigation of how longitudinal care patterns may inform musculoskeletal care characterization and hypothesis generation. It paves the way for future studies examining whether tailored interventions can improve outcomes across distinct patient subgroups.

## Supplementary Information

Below is the link to the electronic supplementary material.

Supplementary Material 1

## Data Availability

This study used data from the *All of Us* Research Program’s Controlled Tier Dataset [version 8], available to authorized users on the Researcher Workbench.

## Abbreviations

ARI: Adjusted rand index
DTW: Dynamic time warping
EHR: Electronic health records
ERAS: Enhanced recovery after surgery
MSD: Musculoskeletal disorders
QoL: Quality of life
TASC: Time-aware sequence clustering
TKR: Total knee replacement
WED: Weighted edit distance
