# Supplementary material for: TASC: a time-aware sequence clustering framework with uncertainty quantification for electronic health record trajectories
Source: BioData Min. 2026 Jun 4;19:60. doi: 10.1186/s13040-026-00569-7 (PMC13455125; doi:10.1186/s13040-026-00569-7)
Supplement: Supplementary file 1 [file 13040_2026_569_MOESM1_ESM.docx]

**SUPPLEMENTARY** **MATERIALS**

**Time gap token construction**

**Supplementary Figure 1**

To explicitly encode temporal spacing between successive diagnosis events, we transformed continuous inter-event time gaps into discrete time-gap tokens (G1–G10). For each patient, we computed the number of days elapsed between consecutive diagnosis events. As shown in Supplementary Figure 1, the distribution of inter-event time gaps across all patients was highly right-skewed, with short intervals and a long tail reflecting prolonged gaps between encounters. Directly modeling raw time differences would therefore disproportionately weight extreme values and reduce robustness in sequence similarity comparisons.

To address this, we discretized inter-event time gaps using cohort-level empirical quantiles. Specifically, time gaps were binned into ten categories (G1–G10) based on deciles of the observed inter-event gap distribution across all patients. Shorter time gaps correspond to lower-index tokens (e.g., G1), while progressively longer gaps map to higher-index tokens (e.g., G10). This quantile-based discretization preserves relative temporal ordering while mitigating sensitivity to outliers and variable follow-up duration. Below is a more detailed description of the range of each decile:

- G1: 0-5 days
- G2: 6-16 days
- G3: 17-34 days
- G4: 35-63 days
- G5: 64-105 days
- G6: 106-174 days
- G7: 175-280 days
- G8: 281-446 days
- G9: 447-744 days
- G10: 744-1470 days
- G_outlier: > 1470 days

**Quality of life index construction**

**Supplementary Table 1. Five Dimensions of Quality of Index by Cluster.** Statistical significance is denoted as P < 0.05 (*), P < 0.01 (**), P < 0.001 (***), and P < 0.0001 (****).

| **Cluster** | **Mobility****  **(walking)** | **Self Care**  **(washing/dressing)** | **Usual Activities****  **(work, study, errands)** | **Pain****  **(pain or discomfort)** | **Emotion****  **(anxiety or depression)** |
| --- | --- | --- | --- | --- | --- |
| 0 (N = 310)  Fast progression to surgery | 2.17 (1.53) | 1.68 (1.12) | 2.27 (1.04) | 2.77 (1.11) | 2.56 (0.88) |
| 1 (N = 210)  Joint pain, obesity | 2.26 (1.56) | 1.72 (1.13) | 2.24 (1.01) | 2.63 (1.07) | 2.43 (0.87) |
| 2 (N = 258)  Joint pain, fracture | 2.32 (1.51) | 1.78 (1.14) | 2.38 (1.03) | 2.85 (1.05) | **2.70 (0.85)** |
| 3 (N = 606)  Soft-tissue dominant | 2.45 (1.55) | 1.87 (1.20) | 2.39 (0.98) | 2.76 (1.04) | 2.50 (0.88) |
| 4 (N = 668)  Complex, chronic | **2.57 (1.54)** | **1.88 (1.15)** | **2.48 (1.02)** | **2.92 (1.12)** | 2.55 (0.91) |

Quality of life was assessed using self-reported responses from the All of Us surveys that map directly onto the five dimensions of the EQ-5D-5L descriptive system, including mobility, self-care, usual activities, pain/discomfort, and anxiety/depression. Each dimension consists of five ordered levels of severity, ranging from “no problems” (level 1) to “extreme problems” (level 5), as defined in the EQ-5D-5L User Guide [1].

For each participant, we extracted their latest available survey response within the 10-year post-surgery window (with a 3-month washout after the index surgery) and assigned numeric scores reflecting the severity level in each dimension. This produced a five-component profile per participant:

- Mobility (difficulty walking)
- Self-care (washing or dressing)
- Usual activities (work, study, household, family, errands)
- Pain/discomfort (subjective pain score)
- Anxiety/depression (self-reported emotional health)

Consistent with EQ-5D-5L scoring conventions, these values do not represent cardinal distances but ordered categories used for descriptive health profiling rather than valuation.

To summarize patient-reported health by cluster, we calculated the mean and standard deviation for each domain within each trajectory subtype, yielding a cluster-level quality-of-life index across the five dimensions. Higher scores indicate greater severity of problems in that domain (range: 1 to 5). This allowed cross-cluster comparisons of functional status, pain burden, and emotional well-being, contextualizing differences in pre-surgical pathways with post-surgical patient-reported health.

In addition to reporting raw domain scores, we calculated a **summary EQ-5D-5L Index value** for each participant using the U.S. time trade-off (TTO) valuation set developed by Pickard et al. (2019). Each of the five EQ-5D-5L dimensions—mobility, self-care, usual activities, pain/discomfort, and anxiety/depression—was mapped to its corresponding **dimension-specific disutility** using published U.S. preference weights. For each dimension, levels 2–5 contribute increasing disutility relative to the level-1 baseline (no problems), as defined by the U.S. TTO scoring system. Disutility for mobility, self-care, usual activities, pain, and anxiety/depression were assigned according to the published look-up tables and then summed to obtain an individual-level **total disutility score**. The EQ-5D-5L Index was computed as:

$$\text{EQ-5D Index}=1-\sum_{\text{dimensions}} \text{disutility}_{d},$$

with higher values indicating better health-related quality of life. This scoring approach reflects population preferences for health states in the United States and provides a standardized measure for comparing quality of life across the trajectory-defined clusters.

References:

1. EuroQol Research Foundation. EQ-5D-5L User Guide. 2015.

2. Pickard AS, Law EH, Jiang R, Pullenayegum E, Shaw JW, Xie F, Oppe M, Boye KS, Chapman RH, Gong CL, Balch A, Busschbach JJV. United States Valuation of EQ-5D-5L Health States Using an International Protocol. Value Health. 2019 Aug;22(8):931-941.

**Frequency-based pattern mining**

To further assess the internal coherence of the trajectory-defined clusters, we conducted frequent-pattern mining as a secondary validation analysis. Patterns were extracted using a support threshold of at least 0.05, meaning that a co-occurring diagnostic set appeared in ≥5% of patients within a cluster, and a lift requirement of >1.5, indicating that diagnoses co-occurred at least 1.5 times more often than expected under independence.

$$support (X)= \frac{Number of patterns containing X}{Total number of patterns}$$

$$lift (X\to Y)= \frac{support (X \cup Y)}{support \left( X \right) \times support (Y)}$$

Under these criteria, Cluster 0 yielded no stable patterns, consistent with its limited number of diagnostic events and rapid progression to surgery. Cluster 1 showed two dominant motifs: (1) co-occurrence of obesity and lower-limb osteoarthritis, and (2) joint-disorder groupings involving lower-limb joint disorder, joint disorder, and osteoarthritis. Cluster 2 demonstrated a frequent set combining enthesopathies, osteoarthritis, lower-limb osteoarthritis, and joint disorder. Cluster 3 exhibited repeated co-occurrence of soft-tissue disorders, lower-limb osteoarthritis, and enthesopathies, reflecting its soft-tissue–dominant trajectory. Cluster 4 displayed the largest and most heterogeneous co-occurring sets, including combinations of osteoarthritis, spondylosis, muscle disorder, bone disorder, knee osteoarthritis, back pain, and soft-tissue disorder. These diagnostic motifs reinforce the differentiation between clusters and highlight clinically meaningful patterns of co-occurring MSD-related conditions within each subgroup.

**Supplementary Table 2. Co-occurring diagnostic event patterns by Cluster**

| **Cluster #** | **Co-occurring patterns (without temporal order)** |
| --- | --- |
| 0 (N = 310): fast progression to surgery | None |
| 1 (N = 210): joint pain, obesity | (obesity, lower_limb_osteoarthritis)  (lower_limb_joint_disorder, lower_limb_osteoarthritis, joint_disorder, osteoarthritis) |
| 2 (N = 258): joint pain, fracture | (enthesopathies, lower_limb_osteoarthritis, osteoarthritis, joint_disorder) |
| 3 (N = 606): soft-tissue dominant | (soft_tissue_disorder, lower_limb_osteoarthritis, joint_disorder)  (enthesopathies, joint_disorder, lower_limb_osteoarthritis) |
| 4 (N = 668): complex, chronic | (bone_disorder, osteoarthritis, spondylosis, muscle_disorder, knee_osteoarthritis, lower_limb_osteoarthritis)  (back_pain, osteoarthritis, spondylosis, muscle_disorder, soft_tissue_disorder, lower_limb_osteoarthritis,) |

**Diagnostic event grouping and distribution**

To construct clinically meaningful event sequences, we grouped individual diagnosis codes into semantically coherent musculoskeletal categories. Each record included the standard concept name, the corresponding source code (ICD-9-CM or ICD-10-CM), its vocabulary designation, and an assigned clinical group (see table below for details).

Groupings were developed through a multistep process. First, we reviewed the hierarchical organization of the ICD musculoskeletal and injury chapters, allowing related diagnoses to be aggregated at a consistent clinical level (e.g., knee osteoarthritis, hip osteoarthritis, and generalized osteoarthritis mapped to broader osteoarthritis categories). Second, we used standard clinical taxonomies and terminology systems to identify semantic similarity among conditions, such as distinctions between joint disorders, soft-tissue disorders, spine-related conditions, metabolic bone disease, and acute injuries. Third, ICD-9 and ICD-10 codes referring to the same underlying pathology were consolidated under a unified standard concept to ensure vocabulary harmonization across coding systems.

Each diagnosis concept was then assigned to one of the predefined musculoskeletal event groups. Concepts with overlapping clinical meaning (e.g., “lower limb joint disorder,” “knee osteoarthritis,” “joint disorder”) were merged into higher-level groupings to reduce redundancy and better reflect pathway-level patterns. Low-frequency diagnoses were reviewed individually, and when clinically appropriate, were merged with the nearest semantic neighbor (e.g., localized adiposity incorporated into obesity grouping if infrequent). All group assignments were iteratively refined through manual review and were validated by clinical experts in musculoskeletal medicine to ensure that category definitions accurately reflected musculoskeletal pathophysiology and real-world care patterns. The resulting consolidated groups served as the event vocabulary for constructing longitudinal diagnosis sequences used in the trajectory clustering analysis.

|  | **Standard concept name** | **Code** | **Vocabulary** | **Group** |
| --- | --- | --- | --- | --- |
| **0** | Open bimalleolar fracture | 824.5 | ICD9CM | ankle_fracture |
| **1** | Open fracture of lateral malleolus | 824.3 | ICD9CM | ankle_fracture |
| **2** | Open fracture of ankle | 824.9 | ICD9CM | ankle_fracture |
| **3** | Fracture of ankle | 824 | ICD9CM | ankle_fracture |
| **4** | Open fracture of medial malleolus | 824.1 | ICD9CM | ankle_fracture |
| **5** | Open trimalleolar fracture | 824.7 | ICD9CM | ankle_fracture |
| **6** | Closed fracture of ankle | 824.8 | ICD9CM | ankle_fracture |
| **7** | Disorder of thoracic spine | M54.15 | ICD10CM | back_pain |
| **8** | Disorder of lumbar spine | M54.15 | ICD10CM | back_pain |
| **9** | Spinal stenosis of thoracic region | 724.01 | ICD9CM | back_pain |
| **10** | Low back pain | 724.2 | ICD9CM | back_pain |
| **11** | Lumbago with sciatica | M54.41 | ICD10CM | back_pain |
| **12** | Peripheral neuritis | 724.4 | ICD9CM | back_pain |
| **13** | Disorder of back | 724.8 | ICD9CM | back_pain |
| **14** | Neurogenic claudication | 724.03 | ICD9CM | back_pain |
| **15** | Pain in spine | M54 | ICD10CM | back_pain |
| **16** | Pain in thoracic spine | 724.1 | ICD9CM | back_pain |
| **17** | Spinal stenosis | 724 | ICD9CM | back_pain |
| **18** | Thoracic nerve root pain | 724.4 | ICD9CM | back_pain |
| **19** | Sciatica | 724.3 | ICD9CM | back_pain |
| **20** | Vertebrogenic pain syndrome | M54.51 | ICD10CM | back_pain |
| **21** | Cervical radiculopathy | M54.12 | ICD10CM | back_pain |
| **22** | Panniculitis affecting sacrum | M54.08 | ICD10CM | back_pain |
| **23** | Hypermobility of coccyx | 724.71 | ICD9CM | back_pain |
| **24** | Lumbosacral radiculopathy | M54.17 | ICD10CM | back_pain |
| **25** | Disorder of cervical spine | M54.13 | ICD10CM | back_pain |
| **26** | Spinal stenosis of lumbar region | 724.02 | ICD9CM | back_pain |
| **27** | Backache | 724.5 | ICD9CM | back_pain |
| **28** | Cervico-occipital neuralgia | M54.81 | ICD10CM | back_pain |
| **29** | Disorder of coccyx | 724.79 | ICD9CM | back_pain |
| **30** | Lumbar radiculopathy | M54.16 | ICD10CM | back_pain |
| **31** | Disorder of sacrum | 724.6 | ICD9CM | back_pain |
| **32** | Thoracic radiculopathy | M54.14 | ICD10CM | back_pain |
| **33** | Disorder of lower leg | M85.862 | ICD10CM | bone_disorder |
| **34** | Disorder of thigh | M85.859 | ICD10CM | bone_disorder |
| **35** | Pathological fracture of neck of femur | 733.14 | ICD9CM | bone_disorder |
| **36** | Stress fracture of fibula | 733.93 | ICD9CM | bone_disorder |
| **37** | Aseptic necrosis of bone | 733.4 | ICD9CM | bone_disorder |
| **38** | Stress fracture | 733.95 | ICD9CM | bone_disorder |
| **39** | Senile osteoporosis | 733.01 | ICD9CM | bone_disorder |
| **40** | Stress fracture of metatarsal bone | 733.94 | ICD9CM | bone_disorder |
| **41** | Pathological fracture | 733.19 | ICD9CM | bone_disorder |
| **42** | Aseptic necrosis of head AND/OR neck of femur | 733.42 | ICD9CM | bone_disorder |
| **43** | Bone cyst | 733.29 | ICD9CM | bone_disorder |
| **44** | Chondromalacia | 733.92 | ICD9CM | bone_disorder |
| **45** | Nonunion of fracture | 733.82 | ICD9CM | bone_disorder |
| **46** | Fracture malunion | 733.81 | ICD9CM | bone_disorder |
| **47** | Osteoporosis | 733 | ICD9CM | bone_disorder |
| **48** | Disorder of bone | M85.80 | ICD10CM | bone_disorder |
| **49** | Pathological fracture of vertebra | 733.13 | ICD9CM | bone_disorder |
| **50** | Pathological fracture of femur | 733.15 | ICD9CM | bone_disorder |
| **51** | Stress fracture of neck of femur | 733.96 | ICD9CM | bone_disorder |
| **52** | Idiopathic osteoporosis | 733.02 | ICD9CM | bone_disorder |
| **53** | Aseptic necrosis of talus | 733.44 | ICD9CM | bone_disorder |
| **54** | Tietze's disease | 733.6 | ICD9CM | bone_disorder |
| **55** | Solitary bone cyst | M85.451 | ICD10CM | bone_disorder |
| **56** | Pathological fracture - lower leg | 733.16 | ICD9CM | bone_disorder |
| **57** | Arrest of bone development AND/OR growth | 733.91 | ICD9CM | bone_disorder |
| **58** | Osteitis condensans | 733.5 | ICD9CM | bone_disorder |
| **59** | Hyperostosis of skull | 733.3 | ICD9CM | bone_disorder |
| **60** | Disorder of musculoskeletal system | 733 | ICD9CM | bone_disorder |
| **61** | Pathological fracture of distal radius and dis... | 733.12 | ICD9CM | bone_disorder |
| **62** | Aseptic necrosis of bone of jaw | 733.45 | ICD9CM | bone_disorder |
| **63** | Aneurysmal bone cyst | M85.50 | ICD10CM | bone_disorder |
| **64** | Monostotic fibrous dysplasia | M85.00 | ICD10CM | bone_disorder |
| **65** | Disuse osteoporosis | 733.03 | ICD9CM | bone_disorder |
| **66** | Stress fracture of shaft of femur | 733.97 | ICD9CM | bone_disorder |
| **67** | Pathological fracture - shoulder | 733.11 | ICD9CM | bone_disorder |
| **68** | Aseptic necrosis of head of humerus | 733.41 | ICD9CM | bone_disorder |
| **69** | Disorder of upper arm | M85.842 | ICD10CM | bone_disorder |
| **70** | Disorder of bone and articular cartilage | 733.9 | ICD9CM | bone_disorder |
| **71** | Disorder of forearm | M85.832 | ICD10CM | bone_disorder |
| **72** | Stress fracture of pelvis | 733.98 | ICD9CM | bone_disorder |
| **73** | Complex regional pain syndrome type I | 733.7 | ICD9CM | bone_disorder |
| **74** | Aseptic necrosis of medial femoral condyle | 733.43 | ICD9CM | bone_disorder |
| **75** | Walking difficulty due to multiple sites | 202572005 | SNOMED | difficulty_walking |
| **76** | Difficulty walking | 719.7 | ICD9CM | difficulty_walking |
| **77** | Bicipital tenosynovitis | 726.12 | ICD9CM | enthesopathies |
| **78** | Olecranon bursitis | 726.33 | ICD9CM | enthesopathies |
| **79** | Tibial collateral ligament bursitis | 726.62 | ICD9CM | enthesopathies |
| **80** | Enthesopathy of wrist AND/OR carpus | 726.4 | ICD9CM | enthesopathies |
| **81** | Enthesopathy of knee | 726.6 | ICD9CM | enthesopathies |
| **82** | Enthesopathy of hip region | 726.5 | ICD9CM | enthesopathies |
| **83** | Pes anserinus tendinitis and bursitis | 726.61 | ICD9CM | enthesopathies |
| **84** | Nontraumatic rotator cuff tear | 726.13 | ICD9CM | enthesopathies |
| **85** | Patellar tendonitis | 726.64 | ICD9CM | enthesopathies |
| **86** | Calcific tendinitis of shoulder | 726.11 | ICD9CM | enthesopathies |
| **87** | Rotator cuff tear arthropathy | 726.1 | ICD9CM | enthesopathies |
| **88** | Achilles bursitis | 726.71 | ICD9CM | enthesopathies |
| **89** | Peripheral enthesopathy | 726.8 | ICD9CM | enthesopathies |
| **90** | Peripheral enthesopathies and allied syndromes | 726 | ICD9CM | enthesopathies |
| **91** | Fibular collateral ligament bursitis | 726.63 | ICD9CM | enthesopathies |
| **92** | Adhesive capsulitis of shoulder | 726 | ICD9CM | enthesopathies |
| **93** | Enthesopathy of elbow region | 726.3 | ICD9CM | enthesopathies |
| **94** | Exostosis | 726.91 | ICD9CM | enthesopathies |
| **95** | Medial epicondylitis | 726.31 | ICD9CM | enthesopathies |
| **96** | Prepatellar bursitis | 726.65 | ICD9CM | enthesopathies |
| **97** | Lateral epicondylitis | 726.32 | ICD9CM | enthesopathies |
| **98** | Disorder of shoulder | 726.2 | ICD9CM | enthesopathies |
| **99** | Disorder of bursa of shoulder region | 726.1 | ICD9CM | enthesopathies |
| **100** | Enthesopathy | 726.9 | ICD9CM | enthesopathies |
| **101** | Tibialis tendinitis | 726.72 | ICD9CM | enthesopathies |
| **102** | Calcaneal spur | 726.73 | ICD9CM | enthesopathies |
| **103** | Enthesopathy of ankle AND/OR tarsus | 726.79 | ICD9CM | enthesopathies |
| **104** | Unexplained falls | 404912005 | SNOMED | falls |
| **105** | Late effect of accidental fall | E929.3 | ICD9CM | falls |
| **106** | At high risk for fall | 4.55202E+17 | SNOMED | falls |
| **107** | Falls | R29.6 | ICD10CM | falls |
| **108** | At risk for falls | 129839007 | SNOMED | falls |
| **109** | Recurrent falls | 279992002 | SNOMED | falls |
| **110** | At risk for injury due to fall | 430576002 | SNOMED | falls |
| **111** | Closed supracondylar fracture of femur | 821.23 | ICD9CM | femur_fracture |
| **112** | Open fracture of shaft of femur | 821.11 | ICD9CM | femur_fracture |
| **113** | Closed fracture of base of neck of femur | S72.044A | ICD10CM | femur_fracture |
| **114** | Closed fracture of femur, greater trochanter | S72.114A | ICD10CM | femur_fracture |
| **115** | Open fracture | S72.092B | ICD10CM | femur_fracture |
| **116** | Closed fracture of femur, lesser trochanter | S72.123A | ICD10CM | femur_fracture |
| **117** | Fracture malunion of pelvis and femur | S72.402P | ICD10CM | femur_fracture |
| **118** | Closed fracture distal femur, lateral condyle | S72.422K | ICD10CM | femur_fracture |
| **119** | Separated epiphysis | S72.442A | ICD10CM | femur_fracture |
| **120** | Closed fracture of intracapsular section of femur | S72.012A | ICD10CM | femur_fracture |
| **121** | Closed fracture of femur, lower epiphysis | S72.442A | ICD10CM | femur_fracture |
| **122** | Closed fracture proximal femur, subtrochanteric | S72.22XD | ICD10CM | femur_fracture |
| **123** | Closed fracture of midcervical section of femur | S72.033A | ICD10CM | femur_fracture |
| **124** | Late effect of fracture of neck of femur | S72.001S | ICD10CM | femur_fracture |
| **125** | Closed fracture of shaft of femur | 821.01 | ICD9CM | femur_fracture |
| **126** | Open fracture of neck of femur | S72.001B | ICD10CM | femur_fracture |
| **127** | Fracture of proximal end of femur | S72.091A | ICD10CM | femur_fracture |
| **128** | Closed fracture of neck of right femur | S72.091A | ICD10CM | femur_fracture |
| **129** | Closed fracture distal femur, medial condyle | S72.431A | ICD10CM | femur_fracture |
| **130** | Closed fracture of head of femur | S72.051A | ICD10CM | femur_fracture |
| **131** | Closed fracture | S72.091A | ICD10CM | femur_fracture |
| **132** | Open fracture of femur | 821.1 | ICD9CM | femur_fracture |
| **133** | Fracture of femur | S72 | ICD10CM | femur_fracture |
| **134** | Closed intertrochanteric fracture | S72.142A | ICD10CM | femur_fracture |
| **135** | Malunion of fracture of proximal femur | S72.002P | ICD10CM | femur_fracture |
| **136** | Open fracture distal femur, lateral condyle | S72.422B | ICD10CM | femur_fracture |
| **137** | Open fracture of femur, distal end | S72.402C | ICD10CM | femur_fracture |
| **138** | Closed fracture of femur | 821 | ICD9CM | femur_fracture |
| **139** | Closed fracture of femoral condyle of femur | 821.21 | ICD9CM | femur_fracture |
| **140** | Closed fracture of femur, distal end | 821.29 | ICD9CM | femur_fracture |
| **141** | Open fracture distal femur, medial condyle | S72.432B | ICD10CM | femur_fracture |
| **142** | Closed fracture of neck of femur | S72.009A | ICD10CM | femur_fracture |
| **143** | Sequelae of fracture of femur | S72.92XS | ICD10CM | femur_fracture |
| **144** | Closed fracture middle phalanx, toe | S92.522A | ICD10CM | foot_fracture |
| **145** | Closed fracture of distal phalanx of great toe | S92.424A | ICD10CM | foot_fracture |
| **146** | Closed fracture of second metatarsal bone | S92.321A | ICD10CM | foot_fracture |
| **147** | Closed fracture of fifth metatarsal bone | S92.351A | ICD10CM | foot_fracture |
| **148** | Fracture of phalanx of foot | S92.912G | ICD10CM | foot_fracture |
| **149** | Fracture of calcaneus | S92.001D | ICD10CM | foot_fracture |
| **150** | Closed fracture of talus | S92.101A | ICD10CM | foot_fracture |
| **151** | Open fracture of phalanx of foot | 826.1 | ICD9CM | foot_fracture |
| **152** | Closed fracture of foot | S92.909A | ICD10CM | foot_fracture |
| **153** | Delayed union of fracture of foot | M84.374G | ICD10CM | foot_fracture |
| **154** | Closed fracture of third metatarsal bone | S92.334A | ICD10CM | foot_fracture |
| **155** | Closed fracture of fourth metatarsal bone | S92.345A | ICD10CM | foot_fracture |
| **156** | Delayed union of fracture | S92.912G | ICD10CM | foot_fracture |
| **157** | Closed fracture of phalanx of foot | 826 | ICD9CM | foot_fracture |
| **158** | Closed fracture of great toe | S92.402A | ICD10CM | foot_fracture |
| **159** | Closed fracture proximal phalanx, toe | S92.512A | ICD10CM | foot_fracture |
| **160** | Closed fracture of proximal phalanx of great toe | S92.412A | ICD10CM | foot_fracture |
| **161** | Closed fracture of metatarsal bone | S92.309A | ICD10CM | foot_fracture |
| **162** | Fracture malunion - ankle and/or foot | S92.502P | ICD10CM | foot_fracture |
| **163** | Fracture of great toe | S92.422D | ICD10CM | foot_fracture |
| **164** | Fracture of foot | S92 | ICD10CM | foot_fracture |
| **165** | Closed fracture distal phalanx, toe | S92.532A | ICD10CM | foot_fracture |
| **166** | Fracture of tarsal bone | S92.202K | ICD10CM | foot_fracture |
| **167** | Closed fracture of calcaneus | S92.002A | ICD10CM | foot_fracture |
| **168** | Closed fracture of cuboid bone of foot | S92.212A | ICD10CM | foot_fracture |
| **169** | Open fracture of calcaneus | S92.001B | ICD10CM | foot_fracture |
| **170** | Closed fracture talus, neck | S92.114A | ICD10CM | foot_fracture |
| **171** | Open fracture of metatarsal bone | S92.301B | ICD10CM | foot_fracture |
| **172** | Metatarsal bone fracture | S92.301D | ICD10CM | foot_fracture |
| **173** | Closed fracture calcaneus, intra-articular | S92.062A | ICD10CM | foot_fracture |
| **174** | Open fracture distal phalanx, toe | S92.532B | ICD10CM | foot_fracture |
| **175** | Stress fracture of foot | M84.376A | ICD10CM | foot_fracture |
| **176** | Open fracture of fifth metatarsal bone | S92.352B | ICD10CM | foot_fracture |
| **177** | Open fracture proximal phalanx, toe | S92.515B | ICD10CM | foot_fracture |
| **178** | Fracture of talus | S92.154D | ICD10CM | foot_fracture |
| **179** | Open fracture of first metatarsal bone | S92.311B | ICD10CM | foot_fracture |
| **180** | Closed fracture talus, body | S92.124A | ICD10CM | foot_fracture |
| **181** | Closed fracture of lateral cuneiform bone of foot | S92.221A | ICD10CM | foot_fracture |
| **182** | Stress fracture of ankle | M84.372A | ICD10CM | foot_fracture |
| **183** | Fracture of metacarpal bone | S92.325G | ICD10CM | foot_fracture |
| **184** | Open fracture of cuboid bone of foot | S92.212B | ICD10CM | foot_fracture |
| **185** | Fracture of cuboid | S92.215D | ICD10CM | foot_fracture |
| **186** | Closed fracture of tarsal bone | S92.202A | ICD10CM | foot_fracture |
| **187** | Fracture of sacrum | S32.10XD | ICD10CM | hip_fracture |
| **188** | Closed fracture pubis | S32.591A | ICD10CM | hip_fracture |
| **189** | Fracture of lumbar spine and/or pelvis | S32 | ICD10CM | hip_fracture |
| **190** | Fracture of pubis | S32.591D | ICD10CM | hip_fracture |
| **191** | Closed fracture of coccyx | S32.2XXA | ICD10CM | hip_fracture |
| **192** | Closed fracture of bones of trunk | S32.9XXA | ICD10CM | hip_fracture |
| **193** | Fracture of ischium | S32.601D | ICD10CM | hip_fracture |
| **194** | Fracture of second lumbar vertebra | S32.020A | ICD10CM | hip_fracture |
| **195** | Closed fracture acetabulum, posterior column | S32.442A | ICD10CM | hip_fracture |
| **196** | Closed fracture of fourth lumbar vertebra | S32.049A | ICD10CM | hip_fracture |
| **197** | Complete disruption of pelvic ring | S32.810D | ICD10CM | hip_fracture |
| **198** | Closed fracture sacrum | S32.10XA | ICD10CM | hip_fracture |
| **199** | Fracture of lumbar spine | S32.009K | ICD10CM | hip_fracture |
| **200** | Multiple closed fractures of pelvis with disru... | S32.810A | ICD10CM | hip_fracture |
| **201** | Closed fracture of ilium | S32.309A | ICD10CM | hip_fracture |
| **202** | Closed fracture lumbar vertebra, burst | S32.001A | ICD10CM | hip_fracture |
| **203** | Fracture of third lumbar vertebra | S32.030G | ICD10CM | hip_fracture |
| **204** | Multiple open fractures of pelvis with disrupt... | 808.53 | ICD9CM | hip_fracture |
| **205** | Multiple pelvic fractures | S32.82XA | ICD10CM | hip_fracture |
| **206** | Sequelae of injuries of neck and trunk | S32.402S | ICD10CM | hip_fracture |
| **207** | Fracture of acetabulum | S32.402D | ICD10CM | hip_fracture |
| **208** | Closed fracture acetabulum, anterior column | S32.432A | ICD10CM | hip_fracture |
| **209** | Fracture of superior pubic ramus | S32.511D | ICD10CM | hip_fracture |
| **210** | Closed fracture of posterior wall of acetabulum | S32.422A | ICD10CM | hip_fracture |
| **211** | Closed fracture of ischium | 808.42 | ICD9CM | hip_fracture |
| **212** | Fracture of ilium | S32.301D | ICD10CM | hip_fracture |
| **213** | Closed Malgaigne fracture | S32.511A | ICD10CM | hip_fracture |
| **214** | Closed fracture of anterior wall of acetabulum | S32.414A | ICD10CM | hip_fracture |
| **215** | Fracture of pelvis | S32.89XD | ICD10CM | hip_fracture |
| **216** | Closed fracture of first lumbar vertebra | S32.018A | ICD10CM | hip_fracture |
| **217** | Fracture of fifth lumbar vertebra | S32.050A | ICD10CM | hip_fracture |
| **218** | Closed fracture of fifth lumbar vertebra | S32.058A | ICD10CM | hip_fracture |
| **219** | Fracture of bones of trunk | S32.9XXD | ICD10CM | hip_fracture |
| **220** | Late effect of fracture of lumbar vertebra | S32.039S | ICD10CM | hip_fracture |
| **221** | Closed fracture lumbar vertebra, wedge | S32.030A | ICD10CM | hip_fracture |
| **222** | Open fracture of pelvis | 808.9 | ICD9CM | hip_fracture |
| **223** | Closed fracture lumbar vertebra | S32.009A | ICD10CM | hip_fracture |
| **224** | Fracture of fourth lumbar vertebra | S32.040D | ICD10CM | hip_fracture |
| **225** | Closed fracture of third lumbar vertebra | S32.038A | ICD10CM | hip_fracture |
| **226** | Closed fracture of medial wall of acetabulum | S32.475A | ICD10CM | hip_fracture |
| **227** | Closed fracture of pelvis | 808.8 | ICD9CM | hip_fracture |
| **228** | Fracture of first lumbar vertebra | S32.010D | ICD10CM | hip_fracture |
| **229** | Wedge fracture of lumbar vertebra | S32.010D | ICD10CM | hip_fracture |
| **230** | Closed fracture of acetabulum | 808 | ICD9CM | hip_fracture |
| **231** | Lower back injury | S32.9XXA | ICD10CM | hip_fracture |
| **232** | Closed fracture of second lumbar vertebra | S32.029A | ICD10CM | hip_fracture |
| **233** | Joint finding | 719.6 | ICD9CM | joint_disorder |
| **234** | Wrist joint unstable | M25.331 | ICD10CM | joint_disorder |
| **235** | Finding of elbow joint | 719.62 | ICD9CM | joint_disorder |
| **236** | Hemarthrosis of shoulder region | M25.011 | ICD10CM | joint_disorder |
| **237** | Finding of shoulder joint | 719.61 | ICD9CM | joint_disorder |
| **238** | Pain in wrist | M25.53 | ICD10CM | joint_disorder |
| **239** | Hemarthrosis of elbow | 719.12 | ICD9CM | joint_disorder |
| **240** | Effusion of joint of shoulder region | M25.412 | ICD10CM | joint_disorder |
| **241** | Instability of hand joint | M25.342 | ICD10CM | joint_disorder |
| **242** | Finding of finger joint | 719.64 | ICD9CM | joint_disorder |
| **243** | Shoulder stiff | 719.51 | ICD9CM | joint_disorder |
| **244** | Hemarthrosis of wrist | 719.13 | ICD9CM | joint_disorder |
| **245** | Hemarthrosis of multiple sites | 719.19 | ICD9CM | joint_disorder |
| **246** | Palindromic rheumatism | 719.3 | ICD9CM | joint_disorder |
| **247** | Shoulder joint pain | 719.41 | ICD9CM | joint_disorder |
| **248** | Arthralgia of the upper arm | 719.42 | ICD9CM | joint_disorder |
| **249** | Palindromic rheumatism of multiple sites | 719.39 | ICD9CM | joint_disorder |
| **250** | Disorder of joint of shoulder region | 719.91 | ICD9CM | joint_disorder |
| **251** | Multiple joint pain | 719.49 | ICD9CM | joint_disorder |
| **252** | Hemarthrosis of hand | 719.14 | ICD9CM | joint_disorder |
| **253** | Flail joint | M25.20 | ICD10CM | joint_disorder |
| **254** | Hemarthrosis | M25.00 | ICD10CM | joint_disorder |
| **255** | Villonodular synovitis of the hand | 719.24 | ICD9CM | joint_disorder |
| **256** | Arthropathy | M25.9 | ICD10CM | joint_disorder |
| **257** | Finding of wrist joint | 719.63 | ICD9CM | joint_disorder |
| **258** | Injury of shoulder region | M25.711 | ICD10CM | joint_disorder |
| **259** | Joint stiffness | M25.60 | ICD10CM | joint_disorder |
| **260** | Osteophyte of bone | M25.78 | ICD10CM | joint_disorder |
| **261** | Wrist joint effusion | 719.03 | ICD9CM | joint_disorder |
| **262** | Effusion of joint of multiple sites | 719.09 | ICD9CM | joint_disorder |
| **263** | Effusion of joint | M25.40 | ICD10CM | joint_disorder |
| **264** | Disorder of wrist | M25.732 | ICD10CM | joint_disorder |
| **265** | Pigmented villonodular synovitis | 719.2 | ICD9CM | joint_disorder |
| **266** | Multiple stiff joints | 719.59 | ICD9CM | joint_disorder |
| **267** | Elbow stiff | M25.629 | ICD10CM | joint_disorder |
| **268** | Wrist stiff | 719.53 | ICD9CM | joint_disorder |
| **269** | Polyarthropathy | 719.89 | ICD9CM | joint_disorder |
| **270** | Wrist joint pain | 719.43 | ICD9CM | joint_disorder |
| **271** | Joint pain | 719.46 | ICD9CM | joint_disorder |
| **272** | Hand joint stiff | 719.54 | ICD9CM | joint_disorder |
| **273** | Elbow joint unstable | M25.321 | ICD10CM | joint_disorder |
| **274** | Shoulder joint unstable | M25.311 | ICD10CM | joint_disorder |
| **275** | Palindromic rheumatism of shoulder region | 719.31 | ICD9CM | joint_disorder |
| **276** | Villonodular synovitis of the shoulder region | 719.21 | ICD9CM | joint_disorder |
| **277** | Effusion of joint of hand | 719.04 | ICD9CM | joint_disorder |
| **278** | Villonodular synovitis of the lower leg | 719.26 | ICD9CM | joint_disorder |
| **279** | Pain in elbow | M25.52 | ICD10CM | joint_disorder |
| **280** | Injury of elbow | M25.722 | ICD10CM | joint_disorder |
| **281** | Elbow joint pain | 719.42 | ICD9CM | joint_disorder |
| **282** | Elbow joint effusion | 719.02 | ICD9CM | joint_disorder |
| **283** | Hand joint pain | 719.44 | ICD9CM | joint_disorder |
| **284** | Villonodular synovitis of wrist | 719.23 | ICD9CM | joint_disorder |
| **285** | Closed fracture of shaft of tibia | S82.252A | ICD10CM | lower_leg_fracture |
| **286** | Open fracture distal tibia | S82.391H | ICD10CM | lower_leg_fracture |
| **287** | Open pilon fracture | S82.872B | ICD10CM | lower_leg_fracture |
| **288** | Closed fracture proximal tibia, lateral condyl... | S82.121A | ICD10CM | lower_leg_fracture |
| **289** | Closed osteochondral fracture of patella | S82.012A | ICD10CM | lower_leg_fracture |
| **290** | Closed fracture proximal tibia, medial condyle... | S82.131A | ICD10CM | lower_leg_fracture |
| **291** | Closed fracture of upper end of fibula | S82.863A | ICD10CM | lower_leg_fracture |
| **292** | Closed fracture patella, comminuted (stellate) | S82.045D | ICD10CM | lower_leg_fracture |
| **293** | Closed fracture proximal tibia, bicondylar | S82.141A | ICD10CM | lower_leg_fracture |
| **294** | Open fracture patella, comminuted (stellate) | S82.042B | ICD10CM | lower_leg_fracture |
| **295** | Closed fracture of distal fibula | S82.821A | ICD10CM | lower_leg_fracture |
| **296** | Open fracture proximal tibia, lateral condyle ... | S82.122B | ICD10CM | lower_leg_fracture |
| **297** | Open fracture proximal tibia, bicondylar | S82.141C | ICD10CM | lower_leg_fracture |
| **298** | Closed fracture of fibula | S82.832A | ICD10CM | lower_leg_fracture |
| **299** | Closed fracture of shaft of fibula | S82.402D | ICD10CM | lower_leg_fracture |
| **300** | Open fracture of shaft of fibula | S82.451B | ICD10CM | lower_leg_fracture |
| **301** | Open fracture of shaft of tibia | S82.251C | ICD10CM | lower_leg_fracture |
| **302** | Closed fracture of condyle of tibia | S82.125A | ICD10CM | lower_leg_fracture |
| **303** | Closed fracture of lateral malleolus | S82.61XD | ICD10CM | lower_leg_fracture |
| **304** | Closed fracture of lower leg | S82.891A | ICD10CM | lower_leg_fracture |
| **305** | Closed fracture distal tibia | S82.302K | ICD10CM | lower_leg_fracture |
| **306** | Open fracture intercondylar spine of tibia | S82.112B | ICD10CM | lower_leg_fracture |
| **307** | Closed fracture of patella | S82.009A | ICD10CM | lower_leg_fracture |
| **308** | Closed fracture patella, transverse | S82.034A | ICD10CM | lower_leg_fracture |
| **309** | Closed fracture of medial malleolus | S82.54XA | ICD10CM | lower_leg_fracture |
| **310** | Closed fracture intercondylar spine of tibia | S82.112A | ICD10CM | lower_leg_fracture |
| **311** | Closed fracture patella, vertical | S82.024A | ICD10CM | lower_leg_fracture |
| **312** | Fracture of lower leg | S82 | ICD10CM | lower_leg_fracture |
| **313** | Closed bimalleolar fracture | S82.841D | ICD10CM | lower_leg_fracture |
| **314** | Closed fracture of upper end of tibia | S82.102A | ICD10CM | lower_leg_fracture |
| **315** | Open fracture of lower leg | S82.892B | ICD10CM | lower_leg_fracture |
| **316** | Fracture malunion - lower leg | S82.892P | ICD10CM | lower_leg_fracture |
| **317** | Closed trimalleolar fracture | S82.851A | ICD10CM | lower_leg_fracture |
| **318** | Open fracture of patella | S82.091B | ICD10CM | lower_leg_fracture |
| **319** | Open fracture of fibula | S82.832B | ICD10CM | lower_leg_fracture |
| **320** | Late effect of fracture of lower extremities | 905.4 | ICD9CM | lower_limb_fracture |
| **321** | Knee joint finding | 719.66 | ICD9CM | lower_limb_joint_disorder |
| **322** | Effusion of joint of pelvic region | 719.05 | ICD9CM | lower_limb_joint_disorder |
| **323** | Osteophyte of left knee | M25.762 | ICD10CM | lower_limb_joint_disorder |
| **324** | Pain of left knee region | M25.562 | ICD10CM | lower_limb_joint_disorder |
| **325** | Pain of right knee region | M25.561 | ICD10CM | lower_limb_joint_disorder |
| **326** | Pain of knee region | M25.569 | ICD10CM | lower_limb_joint_disorder |
| **327** | Bone spur of left foot | M25.775 | ICD10CM | lower_limb_joint_disorder |
| **328** | Flail hip | M25.259 | ICD10CM | lower_limb_joint_disorder |
| **329** | Palindromic rheumatism of pelvic region and thigh | 719.35 | ICD9CM | lower_limb_joint_disorder |
| **330** | Disorder of hip | M25.752 | ICD10CM | lower_limb_joint_disorder |
| **331** | Hip pain | M25.551 | ICD10CM | lower_limb_joint_disorder |
| **332** | Ankle stiff | M25.671 | ICD10CM | lower_limb_joint_disorder |
| **333** | Joint effusion of ankle AND/OR foot | 719.07 | ICD9CM | lower_limb_joint_disorder |
| **334** | Villonodular synovitis of the ankle and foot | 719.27 | ICD9CM | lower_limb_joint_disorder |
| **335** | Bone spur of right foot | M25.774 | ICD10CM | lower_limb_joint_disorder |
| **336** | Bone spur of left ankle | M25.772 | ICD10CM | lower_limb_joint_disorder |
| **337** | Hemarthrosis of knee | M25.062 | ICD10CM | lower_limb_joint_disorder |
| **338** | Villonodular synovitis of the pelvic region an... | 719.25 | ICD9CM | lower_limb_joint_disorder |
| **339** | Hemarthrosis of the ankle | M25.071 | ICD10CM | lower_limb_joint_disorder |
| **340** | Hemarthrosis of the ankle and/or foot | 719.17 | ICD9CM | lower_limb_joint_disorder |
| **341** | Ankle instability | M25.371 | ICD10CM | lower_limb_joint_disorder |
| **342** | Instability of foot joint | M25.374 | ICD10CM | lower_limb_joint_disorder |
| **343** | Pain of right knee joint | M25.561 | ICD10CM | lower_limb_joint_disorder |
| **344** | Foot joint effusion | M25.476 | ICD10CM | lower_limb_joint_disorder |
| **345** | Ankle joint finding | 719.67 | ICD9CM | lower_limb_joint_disorder |
| **346** | Osteophyte of right knee | M25.761 | ICD10CM | lower_limb_joint_disorder |
| **347** | Arthralgia of the ankle and/or foot | 719.47 | ICD9CM | lower_limb_joint_disorder |
| **348** | Knee joint effusion | 719.06 | ICD9CM | lower_limb_joint_disorder |
| **349** | Hip stiff | M25.659 | ICD10CM | lower_limb_joint_disorder |
| **350** | Hip unstable | M25.352 | ICD10CM | lower_limb_joint_disorder |
| **351** | Hip joint effusion | M25.452 | ICD10CM | lower_limb_joint_disorder |
| **352** | Hemarthrosis of hip | 719.15 | ICD9CM | lower_limb_joint_disorder |
| **353** | Ankle joint effusion | M25.473 | ICD10CM | lower_limb_joint_disorder |
| **354** | Disorder of hip joint | 719.95 | ICD9CM | lower_limb_joint_disorder |
| **355** | Arthralgia of the pelvic region and thigh | 719.45 | ICD9CM | lower_limb_joint_disorder |
| **356** | Walking difficulty due to ankle and foot | 719.77 | ICD9CM | lower_limb_joint_disorder |
| **357** | Foot joint stiff | 719.57 | ICD9CM | lower_limb_joint_disorder |
| **358** | Unstable knee | M25.369 | ICD10CM | lower_limb_joint_disorder |
| **359** | Bone spur of right hip | M25.751 | ICD10CM | lower_limb_joint_disorder |
| **360** | Knee stiff | M25.661 | ICD10CM | lower_limb_joint_disorder |
| **361** | Joint pain of pelvic region | 719.45 | ICD9CM | lower_limb_joint_disorder |
| **362** | Degenerative joint disease of pelvis | 715.95 | ICD9CM | lower_limb_osteoarthritis |
| **363** | Arthropathy of knee joint | 715.36 | ICD9CM | lower_limb_osteoarthritis |
| **364** | Disorder of joint of ankle and/or foot | 715.37 | ICD9CM | lower_limb_osteoarthritis |
| **365** | Osteoarthritis of knee | 715.96 | ICD9CM | lower_limb_osteoarthritis |
| **366** | Bilateral osteoarthritis of knees | 715.16 | ICD9CM | lower_limb_osteoarthritis |
| **367** | Arthritis of knee | 715.36 | ICD9CM | lower_limb_osteoarthritis |
| **368** | Arthropathy of pelvis | 715.35 | ICD9CM | lower_limb_osteoarthritis |
| **369** | Primary localized osteoarthrosis of pelvic region | 715.15 | ICD9CM | lower_limb_osteoarthritis |
| **370** | Localized, primary osteoarthritis of the ankle... | M19.072 | ICD10CM | lower_limb_osteoarthritis |
| **371** | Disorder of knee | 715.26 | ICD9CM | lower_limb_osteoarthritis |
| **372** | Secondary localized osteoarthrosis of pelvic r... | 715.25 | ICD9CM | lower_limb_osteoarthritis |
| **373** | Localized, secondary osteoarthritis of the ank... | M19.271 | ICD10CM | lower_limb_osteoarthritis |
| **374** | Degenerative joint disease of ankle AND/OR foot | 715.97 | ICD9CM | lower_limb_osteoarthritis |
| **375** | Paraplegic immobility syndrome | M62.3 | ICD10CM | muscle_disorder |
| **376** | Spasm | M62.838 | ICD10CM | muscle_disorder |
| **377** | Muscle contracture | M62.471 | ICD10CM | muscle_disorder |
| **378** | Muscle atrophy | M62.50 | ICD10CM | muscle_disorder |
| **379** | Muscle weakness | M62.81 | ICD10CM | muscle_disorder |
| **380** | Rhabdomyolysis | M62.82 | ICD10CM | muscle_disorder |
| **381** | Diastasis of muscle | M62.00 | ICD10CM | muscle_disorder |
| **382** | Nontraumatic rupture of muscle | M62.151 | ICD10CM | muscle_disorder |
| **383** | Spasm of back muscles | M62.830 | ICD10CM | muscle_disorder |
| **384** | Diffuse cervicobrachial syndrome | 723.3 | ICD9CM | neck_disorder |
| **385** | Cervical syndrome | 723.8 | ICD9CM | neck_disorder |
| **386** | Panniculitis of neck | 723.6 | ICD9CM | neck_disorder |
| **387** | Torticollis | 723.5 | ICD9CM | neck_disorder |
| **388** | Spinal stenosis in cervical region | 723 | ICD9CM | neck_disorder |
| **389** | Cervical posterior longitudinal ligament ossif... | 723.7 | ICD9CM | neck_disorder |
| **390** | Brachial radiculitis | 723.4 | ICD9CM | neck_disorder |
| **391** | Disorder of neck | 723.8 | ICD9CM | neck_disorder |
| **392** | Musculoskeletal disorder of the neck | 723.9 | ICD9CM | neck_disorder |
| **393** | Cervicocranial syndrome | 723.2 | ICD9CM | neck_disorder |
| **394** | Neck pain | 723.1 | ICD9CM | neck_disorder |
| **395** | Severe obesity | 8.3911E+13 | SNOMED | obesity |
| **396** | Drug-induced obesity | E66.1 | ICD10CM | obesity |
| **397** | Morbid obesity | 278.01 | ICD9CM | obesity |
| **398** | Childhood obesity | 444862003 | SNOMED | obesity |
| **399** | Simple obesity | 415530009 | SNOMED | obesity |
| **400** | Obesity caused by energy imbalance | 722596001 | SNOMED | obesity |
| **401** | Obesity | 278 | ICD9CM | obesity |
| **402** | Extreme obesity with alveolar hypoventilation | 278.03 | ICD9CM | obesity |
| **403** | Obesity in mother complicating childbirth | O99.214 | ICD10CM | obesity |
| **404** | Maternal obesity complicating pregnancy, child... | O99.211 | ICD10CM | obesity |
| **405** | Central obesity | 248311001 | SNOMED | obesity |
| **406** | Localized adiposity | 278.1 | ICD9CM | obesity |
| **407** | Osteoarthritis of wrist | 715.93 | ICD9CM | osteoarthritis |
| **408** | Arthritis of shoulder region joint | 715.31 | ICD9CM | osteoarthritis |
| **409** | Localized, primary osteoarthritis of the hand | M19.041 | ICD10CM | osteoarthritis |
| **410** | Osteoarthrosis of the carpometacarpal joint of... | 715.34 | ICD9CM | osteoarthritis |
| **411** | Localized, primary osteoarthritis of the shoul... | M19.012 | ICD10CM | osteoarthritis |
| **412** | Arthropathy of joint of hand | 715.34 | ICD9CM | osteoarthritis |
| **413** | Localized, primary osteoarthritis of the wrist | 715.13 | ICD9CM | osteoarthritis |
| **414** | Generalized osteoarthritis of the hand | 715.04 | ICD9CM | osteoarthritis |
| **415** | Degenerative joint disease of hand | 715.94 | ICD9CM | osteoarthritis |
| **416** | Arthropathy of elbow | 715.32 | ICD9CM | osteoarthritis |
| **417** | Osteoarthrosis involving multiple sites but no... | 715.89 | ICD9CM | osteoarthritis |
| **418** | Degenerative joint disease of shoulder region | 715.91 | ICD9CM | osteoarthritis |
| **419** | Disorder of wrist joint | 715.33 | ICD9CM | osteoarthritis |
| **420** | Localized osteoarthrosis uncertain if primary ... | 715.37 | ICD9CM | osteoarthritis |
| **421** | Secondary osteoarthritis | M19.93 | ICD10CM | osteoarthritis |
| **422** | Degenerative joint disease involving multiple ... | 715.09 | ICD9CM | osteoarthritis |
| **423** | Post traumatic osteoarthritis | M19.92 | ICD10CM | osteoarthritis |
| **424** | Osteoarthritis of elbow | 715.92 | ICD9CM | osteoarthritis |
| **425** | Idiopathic osteoarthritis | M19.91 | ICD10CM | osteoarthritis |
| **426** | Osteoarthritis | 715.9 | ICD9CM | osteoarthritis |
| **427** | Localized, secondary osteoarthritis of the sho... | M19.212 | ICD10CM | osteoarthritis |
| **428** | Localized, secondary osteoarthritis | 715.26 | ICD9CM | osteoarthritis |
| **429** | Localized, primary osteoarthritis of elbow | M19.022 | ICD10CM | osteoarthritis |
| **430** | Localized, secondary osteoarthritis of the hand | 715.24 | ICD9CM | osteoarthritis |
| **431** | Localized, primary osteoarthritis | 715.16 | ICD9CM | osteoarthritis |
| **432** | Localized osteoporosis - Lequesne | M81.6 | ICD10CM | osteoporosis |
| **433** | Sequela of disorder | M80.00XS | ICD10CM | osteoporotic_fracture |
| **434** | Osteoporotic fracture of vertebra | M80.08XD | ICD10CM | osteoporotic_fracture |
| **435** | Osteoporotic fracture of humerus | M80.021D | ICD10CM | osteoporotic_fracture |
| **436** | Osteoporotic fracture of femur | M80.059A | ICD10CM | osteoporotic_fracture |
| **437** | Osteoporotic fracture | M80.00XD | ICD10CM | osteoporotic_fracture |
| **438** | Pain in right foot | M79.671 | ICD10CM | soft_tissue_disorder |
| **439** | Foot pain | M79.673 | ICD10CM | soft_tissue_disorder |
| **440** | Muscle pain | M79.1 | ICD10CM | soft_tissue_disorder |
| **441** | Musculoskeletal symptom | 729.89 | ICD9CM | soft_tissue_disorder |
| **442** | Pain in left foot | M79.672 | ICD10CM | soft_tissue_disorder |
| **443** | Fat pad syndrome | M79.4 | ICD10CM | soft_tissue_disorder |
| **444** | Cramp in limb | 729.82 | ICD9CM | soft_tissue_disorder |
| **445** | Seroma due to trauma | 729.91 | ICD9CM | soft_tissue_disorder |
| **446** | Hematoma | 729.92 | ICD9CM | soft_tissue_disorder |
| **447** | Fibromyalgia | M79.7 | ICD10CM | soft_tissue_disorder |
| **448** | Pain in right arm | M79.601 | ICD10CM | soft_tissue_disorder |
| **449** | Neuralgia | M79.2 | ICD10CM | soft_tissue_disorder |
| **450** | Finding of head and neck region | M79.12 | ICD10CM | soft_tissue_disorder |
| **451** | Swelling of limb | 729.81 | ICD9CM | soft_tissue_disorder |
| **452** | Hypertrophy of fat pad of knee | 729.31 | ICD9CM | soft_tissue_disorder |
| **453** | Compartment syndrome of lower limb | 729.72 | ICD9CM | soft_tissue_disorder |
| **454** | Pain in left arm | M79.602 | ICD10CM | soft_tissue_disorder |
| **455** | Pain in forearm | M79.632 | ICD10CM | soft_tissue_disorder |
| **456** | Nontraumatic exertional compartment syndrome | 729.79 | ICD9CM | soft_tissue_disorder |
| **457** | Pain in left lower limb | M79.662 | ICD10CM | soft_tissue_disorder |
| **458** | Rheumatism | 729 | ICD9CM | soft_tissue_disorder |
| **459** | Fasciitis | 729.4 | ICD9CM | soft_tissue_disorder |
| **460** | Disorder of soft tissue | 729.9 | ICD9CM | soft_tissue_disorder |
| **461** | Pain in limb | 729.5 | ICD9CM | soft_tissue_disorder |
| **462** | Disorder of muscle | 729.1 | ICD9CM | soft_tissue_disorder |
| **463** | Nontraumatic hematoma of soft tissue | M79.81 | ICD10CM | soft_tissue_disorder |
| **464** | Pain in upper limb | M79.603 | ICD10CM | soft_tissue_disorder |
| **465** | Disorder of hand | M79.2 | ICD10CM | soft_tissue_disorder |
| **466** | Hand pain | M79.642 | ICD10CM | soft_tissue_disorder |
| **467** | Peripheral nerve disease | 729.2 | ICD9CM | soft_tissue_disorder |
| **468** | Pain in lower limb | M79.606 | ICD10CM | soft_tissue_disorder |
| **469** | Pain in right lower limb | M79.661 | ICD10CM | soft_tissue_disorder |
| **470** | Pain in upper arm | M79.62 | ICD10CM | soft_tissue_disorder |
| **471** | Pain in finger | M79.644 | ICD10CM | soft_tissue_disorder |
| **472** | Residual foreign body in soft tissue | 729.6 | ICD9CM | soft_tissue_disorder |
| **473** | Pain in right hand | M79.641 | ICD10CM | soft_tissue_disorder |
| **474** | Pain in toe | M79.674 | ICD10CM | soft_tissue_disorder |
| **475** | Panniculitis | 729.39 | ICD9CM | soft_tissue_disorder |
| **476** | Thigh pain | M79.652 | ICD10CM | soft_tissue_disorder |
| **477** | Compartment syndrome | M79.A9 | ICD10CM | soft_tissue_disorder |
| **478** | Spontaneous hemorrhage | M79.81 | ICD10CM | soft_tissue_disorder |
| **479** | Lumbosacral spondylosis without myelopathy | M47.817 | ICD10CM | spondylosis |
| **480** | Nerve root disorder | M47.20 | ICD10CM | spondylosis |
| **481** | Thoracic spondylosis with myelopathy | M47.14 | ICD10CM | spondylosis |
| **482** | Spondylosis | M47.9 | ICD10CM | spondylosis |
| **483** | Lumbosacral spondylosis | M47.897 | ICD10CM | spondylosis |
| **484** | Thoracic spondylosis | M47.894 | ICD10CM | spondylosis |
| **485** | Cervical spondylosis | M47.892 | ICD10CM | spondylosis |
| **486** | Lumbar spondylosis with myelopathy | M47.26 | ICD10CM | spondylosis |
| **487** | Cervical spondylosis without myelopathy | M47.812 | ICD10CM | spondylosis |
| **488** | Lumbar spondylosis | M47.816 | ICD10CM | spondylosis |
| **489** | Lumbosacral spondylosis with radiculopathy | M47.27 | ICD10CM | spondylosis |
| **490** | Radiculopathy due to cervical spondylosis | M47.22 | ICD10CM | spondylosis |
| **491** | Spondylosis without myelopathy | M47.815 | ICD10CM | spondylosis |
| **492** | Thoracic spondylosis with radiculopathy | M47.24 | ICD10CM | spondylosis |
| **493** | Thoracic spondylosis without myelopathy | M47.814 | ICD10CM | spondylosis |
| **494** | Spinal nerve root finding | M47.25 | ICD10CM | spondylosis |
| **495** | Myelopathy due to cervical spondylosis | M47.12 | ICD10CM | spondylosis |
| **496** | Vitamin D deficiency | 268.9 | ICD9CM | vitamin_d_deficiency |
| **497** | Decreased vitamin D | 131114008 | SNOMED | vitamin_d_deficiency |
| **498** | Disorder of vitamin D | 386068004 | SNOMED | vitamin_d_deficiency |

*Supplementary Figure 1.* ***Diagnosis Event Categories and Distribution***

The distribution of diagnosis events across the cohort is shown in Supplementary Figure 1. The bar graph displays the frequency of each grouped musculoskeletal or comorbidity-related diagnosis after semantic consolidation. Categories are ordered from most to least common, illustrating the prominence of **lower-limb joint disorders**, **general joint disorders**, **lower-limb osteoarthritis**, and **soft-tissue disorders**, followed by **osteoporosis-related pain and fragility conditions**. Less frequent but clinically important diagnoses include **falls**, **osteoporotic fractures**, **localized adiposity**, and various site-specific fractures. This distribution highlights the wide range of musculoskeletal and injury-related events captured in the patient trajectories and the substantial heterogeneity in diagnostic burden prior to total knee replacement.

Based on the grouping process described above, the final set of clinically meaningful diagnosis categories included:

- **Lower-limb joint disorder**
- **Joint disorder (unspecified)**
- **Lower-limb osteoarthritis**
- **Soft-tissue disorder**
- **Osteoarthritis (unspecified)**
- **Back pain**
- **Obesity**
- **Enthesopathies**
- **Bone disorder**
- **Neck disorder**
- **Knee osteoarthritis**
- **Vitamin D deficiency**
- **Spondylosis**
- **Muscle disorder**
- **Osteoporosis**
- **Difficulty walking**
- **Foot fracture**
- **Ankle fracture**
- **Femur fracture**
- **Falls**
- **Lower-leg fracture**
- **Hip fracture**
- **Lower-limb fracture (unspecified)**
- **Osteoporotic fracture**
- **Localized adiposity**

These categories reflect the major musculoskeletal domains present in the cohort, including joint degeneration, soft-tissue conditions, spinal pathology, metabolic bone disease, fragility events, obesity-related diagnoses, and acute injuries, and formed the event vocabulary used to construct all longitudinal patient trajectories.

**Word2Vec embedding training and validation**

To capture semantic relationships among diagnosis-event tokens, we trained a Word2Vec model on the full set of patient trajectories. Each trajectory consisted of an ordered sequence of **diagnosis tokens** interleaved with **time-gap tokens**, allowing the embedding space to encode both clinical meaning and temporal context. The model was trained using the skip-gram architecture (sg = 1), which is well suited for learning representations of sparse medical events because it prioritizes predicting surrounding context tokens from a given focal event. We used a vector size of **30 dimensions**, selected to approximate the average sequence length across patients and provide sufficient capacity to encode clinical variation without overfitting. A context window of **five events** captured medium-range dependencies reflective of typical care patterns in musculoskeletal diagnosis sequences. All tokens were included (min_count = 1) to preserve rare but clinically meaningful diagnoses such as fracture subtypes or metabolic bone disorders. Training was performed for **20 epochs** with a fixed random seed for reproducibility.

The resulting embedding matrix provided a continuous representation of each token in the trajectory vocabulary. Diagnosis–diagnosis substitution costs for the weighted edit-distance metric were computed using **cosine dissimilarity** between their corresponding vectors, enabling clinically similar diagnoses (e.g., soft_tissue_disorder and enthesopathies) to incur lower substitution penalties than semantically distant ones (e.g., lower_limb_osteoarthritis and hip_fracture). Time-gap tokens, which were also embedded jointly with diagnosis tokens, contributed temporal information to the embedding space and enabled mixed substitutions that reflect both timing and semantic proximity.

*Supplementary Figure 2. UMAP visualization of Word2Vec embedding*

To qualitatively assess the structure of the embedding space, we applied **UMAP (Uniform Manifold Approximation and Projection)** to reduce the 30-dimensional Word2Vec vectors to two dimensions (Supplementary Figure 2). The projection revealed coherent clustering of clinically related diagnoses, for example, joint-pain-issue terms (e.g., lower_limb_joint_disorder, knee_osteoarthritis) grouped together, while similar fracture types (e.g., lower_limb_fracture, ankle_fracture) formed a distinct high-severity cluster along with difficulty walking and falls. *Obesity* and vitamin_d_deficiency were positioned apart from acute injuries, reflecting their different semantic roles in patient trajectories. Time-gap tokens (G1–G10) aligned along a gradient, validating that the model learned a monotonic temporal structure without overpowering the clinical semantics. This pattern supports the internal validity of the embeddings and their suitability for use in the subsequent edit-distance–based trajectory clustering.

**Software implementation**

All analyses were conducted in the *All of Us* Researcher Workbench using Python (version 3.10.16) within the JupyterLab environment. Data manipulation, numerical operations, and preprocessing were performed using *NumPy*, *pandas*, and *SciPy*. Visualization of clustering outputs, embedding spaces, and event distributions was carried out using *matplotlib* and *seaborn*.

Trajectory construction and sequence representation were implemented in Python. Word2Vec embeddings were trained using the *gensim* library (skip-gram architecture), and dimensionality reduction for embedding quality assessment was performed with UMAP (via the *umap-learn* package) and t-SNE from scikit-learn. Pairwise trajectory distances were computed using a custom *numba*-accelerated implementation of weighted edit distance combining semantic (Word2Vec-based) substitution costs and time-gap dissimilarity.

Clustering was performed using K-Medoids from the *sklearn-extra* library, selected for its ability to operate directly on precomputed distance matrices. Cluster quality was evaluated using the silhouette score (*scikit-learn*), and robustness was assessed via a subsampling-based stability analysis using the Adjusted Rand Index (ARI). For association analyses, group differences in continuous variables were assessed using one-way ANOVA (*SciPy*), and categorical variables using Chi-square tests, with false-discovery-rate correction via the Benjamini–Hochberg procedure (*statsmodels.stats.multitest*).

Frequent-pattern mining was conducted using *apriori* and *association_rules* from the *mlxtend.frequent_patterns* module to identify high-support, high-lift diagnostic co-occurrence patterns within each trajectory subtype.

All packages were used in their stable versions available in the All of Us cloud environment at the time of analysis. A full list is provided below for reproducibility.

- NumPy: 1.24.4
- pandas: 2.2.3
- SciPy: 1.11.4
- scikit-learn: 1.6.1
- matplotlib: 3.7.3
- seaborn: 0.12.2
- gensim: 4.3.3
- umap-learn: 0.5.7
- sklearn-extra: 0.3.0
- statsmodels: 0.14.4
- mlxtend: 0.23.4
- numba: 0.58.1
- NumPy: 1.24.4
- pandas: 2.2.3
- SciPy: 1.11.4
- scikit-learn: 1.6.1
- matplotlib: 3.7.3
- seaborn: 0.12.2
- gensim: 4.3.3
- umap-learn: 0.5.7
- sklearn-extra: 0.3.0
- statsmodels: 0.14.4
- mlxtend: 0.23.4
- numba: 0.58.1
